# Evaluation the role of cuproptosis-related genes in the pathogenesis, diagnosis and molecular subtypes identification of atherosclerosis

**DOI:** 10.1016/j.heliyon.2023.e21158

**Published:** 2023-10-18

**Authors:** Mengxi Wang, Liying Cheng, Qian Xiang, Ziwei Gao, Yuhan Ding, Haitao Xie, Xiaohu Chen, Peng Yu, Le Shen

**Affiliations:** a Department of Cardiology, Affiliated Hospital of Nanjing University of Chinese Medicine, Nanjing 210029, China; b Department of Cardiology, Jiangsu Province Hospital of Chinese Medicine, Nanjing 210029, China; c First Clinical Medical College, Nanjing University of Chinese Medicine, Nanjing 210023, China; d State Key Laboratory of Component-based Chinese Medicine, Tianjin University of Traditional Chinese Medicine, Tianjin 301617, China

**Keywords:** Cuproptosis, Atherosclerosis, Pathogenesis, Diagnosis, Molecular subtypes

## Abstract

**Background:**

At present, the pathogenesis of atherosclerosis has not been fully elucidated, and the diagnosis and treatment face great challenges. Cuproptosis is a novel cell death pattern that might be involved in the development of atherosclerosis. However, no research has reported the correlation between cuproptosis and atherosclerosis.

**Methods:**

The differential cuproptosis-related genes (CRGs) between atherosclerosis group and control group (A-CRGs) were discovered via differential expression analysis. The correlation analysis, PPI network analysis, GO, KEGG and GSEA analysis were performed to investigate the function of A-CRGs. The differences of biological function between atherosclerosis group and control group were investigated via immune infiltration analysis and GSVA. The LASSO regression, nomogram and machine learning models were constructed to predict atherosclerosis risk. The atherosclerosis molecular subtypes clusters were discovered via unsupervised cluster analysis. Subsequently, we used the above research methods to analyze the differential CRGs between clusters (M-CRGs) and evaluate the molecular subtypes identification performance of M-CRGs. Finally, we verified the diagnostic value for atherosclerosis and role in cuproptosis of these CRGs through the validation set and in vitro experiments.

**Results:**

Five A-CRGs were identified and they were mainly related to the biological function of copper ion metabolism and immune inflammatory response. The diagnostic models and nomogram of atherosclerosis based on 5 A-CRGs indicated that these genes had well diagnostic value. A total of two molecular subtypes clusters were obtained in the atherosclerosis group. There were many differences in biological functions between these two molecular subtypes clusters, such as mitochondrial outer membrane permeabilization and primary immunodeficiency. In addition, 3 M-CRGs were identified in the 2 clusters. Machine learning models and nomogram constructed based on M-CRGs showed that these genes had well molecular subtypes identification efficacy. In the end, the results of in vitro experiment and validation set confirmed the diagnostic value for atherosclerosis and role in cuproptosis of these genes.

**Conclusion:**

The cuproptosis may be a potential pathogenesis of atherosclerosis and CRGs may be promising markers for the diagnosis and molecular subtypes identification of atherosclerosis.

## Graphical abstract

**Figure:**
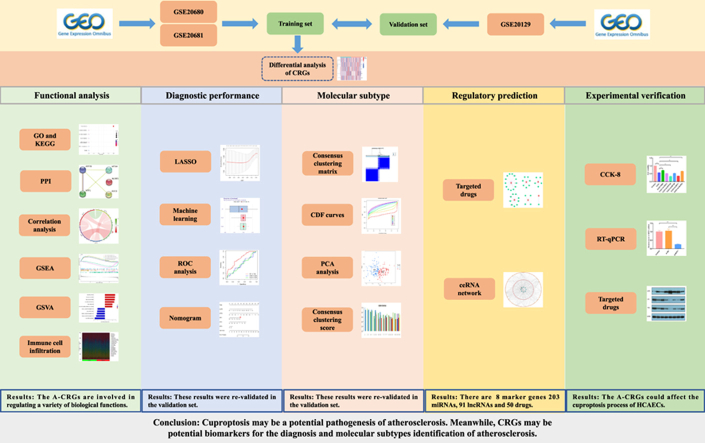

## 1 Introduction

Atherosclerosis is one of the most harmful chronic diseases characterized by a build-up of lipids under the intima of the arteries, which will eventually lead to narrowing of the lumen [1]. The incidence of atherosclerotic cardiovascular diseases remains high, causing a large number of deaths and disabilities each year and placing a heavy burden on society [2]. Recently, the research on the pathophysiology of atherosclerosis has been gradually deepened, but it has not been fully elucidated [3]. Early diagnosis and treatment are important for atherosclerosis. However, the diagnosis of atherosclerosis mainly depends on instrument examination, especially the diagnosis of atherosclerosis in the large artery of the heart and brain requires invasive interventional examination, and there is still a lack of effective blood diagnostic markers [4]. In terms of treatment, although the development of new drugs for atherosclerosis has made remarkable achievements in recent years, there are still a considerable number of patients who have not achieved satisfactory efficacy [5]. The heterogeneity of disease molecular subtypes among different atherosclerosis patients may be the main reason for the difference in treatment effect [6]. It can be seen that the pathogenesis, diagnosis and treatment of atherosclerosis are still facing great challenges, and further investigation is needed to solve the current dilemma.

Cuproptosis is a recently discovered cell death pattern that relies on mitochondrial metabolism and cellular respiration and is characterized by the accumulation of lipoylated proteins and the loss of iron-sulfur cluster proteins [7]. Mitochondrial dysfunction is one of the core pathological processes of cuproptosis. Previous research has shown that both copper levels and mitochondrial function were related to the occurrence and development of atherosclerosis. Mitochondrial dysfunction could affect endothelial cell function and survival, thus accelerating atherosclerosis [8]. The expression levels of serum copper are positively correlated with the risk of atherosclerosis, but the specific mechanism has not been fully explained. Previous researchers thought that it might be related to neointimal formation and immune inflammatory response [9]. Therefore, based on these phenomena found in previous studies, we speculated that cuproptosis may be one kind of the pathogenesis of atherosclerosis. However, no research has reported the correlation between cuproptosis and atherosclerosis, nor the value of cuproptosis-related genes (CRGs) for molecular subtypes identification and diagnosis of atherosclerosis. Therefore, our research attempted to explore the value of CRGs in pathogenesis, diagnosis and molecular subtypes identification of atherosclerosis via bioinformatics analysis and in vitro experiments, in order to provide new insights into the study of atherosclerosis.

## 2 Material and methods

### 2.1 Data access

Three atherosclerosis datasets (GSE20680, GSE20681 and GSE20129) were acquired from the Gene Expression Omnibus (GEO). The GSE20680 and GSE20681 datasets were designated as training set, containing a total of 186 patients with atherosclerosis and 207 control population without atherosclerosis. The GSE20129 dataset was designated as validation set, containing a total of 49 patients with atherosclerosis and 86 control population without atherosclerosis. The baseline characteristics of the participants contained in these 3 datasets were summarized in the Supplementary Tables S1, S2, and S3, respectively. The baseline characteristics information for the GSE20680 and GSE20681 datasets was derived from the descriptions in the original literature of these two datasets [10]. The baseline characteristics information was not reported in the original literature for the GSE20129 dataset [11]. Therefore, the baseline characteristics information of the GSE20129 dataset was derived from data files in the GEO database, which contained only the racial information of the participants. The CRGs were obtained from the published studied and presented in the Supplementary Table S4 [[12], [13], [14], [15]].

### 2.2 Evaluation and elimination of batch effects

We adopted principal component analysis (PCA) to evaluate if batch effects existed between different datasets or between data from different platforms. If batch effects existed, the "sva" package was used to normalize the data, and then the PCA was conducted to assess if batch effects of the processed data are eliminated.

### 2.3 Differential expression analysis of CRGs

First, we extracted the expression data of CRGs from the datasets. Then, we adopted the package “limma” to identify CRGs that were differentially expressed between atherosclerosis and control group (A-CRGs), or CRGs that were differentially expressed between different molecular subtypes clusters (M-CRGs). The *P*-value less than 0.05 was considered be significant differences. Then, the heatmap and boxplot of differential CRGs was drawn by “pheatmap” and “ggpubr” packages. Finally, we used the “RCircos” package to visualize the gene chromosome position.

### 2.4 Gene set variation analysis (GSVA)

We used the package “GSVA” and “limma” to conduct GSVA to identify differential biological functions between different groups. The difference was considered significant when the absolute t value was greater than 2.

### 2.5 Single-gene gene set enrichment analysis (GSEA)

We conducted the single-gene GSEA of the 5 A-CRGs and 3 M-CRGs to investigate the biological processes involved in these genes. First, we assessed the association between these CRGs and all other genes in whole dataset. Then, we sequence the genes in the gene set based on their association. Finally, the significantly enriched biological processes were identified according to GO and KEGG data files.

### 2.6 GO analysis, KEGG analysis and protein–protein interaction analysis

We used the STRING database and “clusterProfiler” package to conduct protein–protein interaction (PPI) analysis, GO analysis and KEGG analysis of CRGs.

### 2.7 Analysis of immune cell infiltration

The immune cell infiltration analysis methods in this study referred to previous literature [16,17]. The differences of relative immune cell infiltration abundance and proportion in each sample between different groups were assessed according to the CIBERSORT algorithm.

### 2.8 Unsupervised clustering for atherosclerosis patients based on A-CRGs

We conducted the unsupervised clustering analysis on atherosclerosis groups based on 5 A-CRGs through “ConsensusClusterPlus” package. The most appropriate number of clusters was determined based on cumulative distribution function (CDF) curves, consensus clustering matrix, consensus clustering score and CDF delta area curves.

### 2.9 Construction and assessment of diagnosis and molecular subtype identification models for atherosclerosis

We used the package “glmnet” to perform the least absolute shrinkage and selection operator (LASSO) regression to assess the performance of 5 A-CRGs and 3 M-CRGs for molecular subtypes identification and diagnosis of atherosclerosis. Then, we adopted the package “caret” to perform 3 machine learning algorithms based on the key genes screened by LASSO regression. These 3 machine learning algorithms included support vector machine model (SVM), random forest model (RF) and generalized linear model (GLM). In the end, we assessed the molecular subtypes identification and diagnosis performance of these 3 machine learning algorithms through residual boxplots, cumulative residual distribution curves and receiver operating characteristic (ROC) curves.

### 2.10 Construction and evaluation of the nomogram

We used the package “rms” to construct the nomogram on the basis of A-CRGs or M-CRGs. Each gene is given a different score based on its level of expression. The sum of these scores indicated the probability of occurrence of a disease or molecular subtype. The predictive power of the nomogram was comprehensively assessed based on Calibration curves and decision curve analysis (DCA).

### 2.11 Prediction of targeted drugs

We predicted drugs that might target binding of these key CRGs using the DGIdb database, and used the software Cytoscape to present the results.

### 2.12 Establishment of the ceRNA network

The miRNAs that might target binding of these key CRGs were identified based on the miRanda, miRDB and TargetScan databases. Meanwhile, lncRNAs that might target binding of these miRNAs were identified based on the lncBase and mircode databases. The software Cytoscape was then employed to establish the ceRNA network of mRNA-miRNA-lncRNA interactions.

### 2.13 Materials and reagents

The antibodies of ferredoxin-1 (FDX1), lipoyl synthase (LIAS) and heat shock protein 70 (HSP70) were purchased from Proteintech Group. The copper chloride (CuCl_2_) and glutathione (GSH) were obtained from Sigma Aldrich. The endothelial cell medium (ECM) was acquired from ScienCell. The siRNAs were bought from Ambion (Ambion Silencer Select siRNA). The transfection reagent Lipofectamine RNAiMAX was purchased from Invitrogen. The real time quantitative polymerase chain reaction (RT-qPCR) reagents (reverse transcription reagents and amplification reagents) were acquired from Yeasen Biotechnology.

### 2.14 Cell culture and model establishment

We cultured the human coronary artery endothelial cells (HCAECs) in ECM which was supplemented with 5 % FBS and 1 % penicillin-treptomycin. The construction of cuproptosis cell model referred to the previously studies and the specific method is as follows [18]. The HCAECs were treated with CuCl_2_ for 24 h. The treatment concentration of CuCl_2_ was determined by the screening results of CCK-8 cell proliferation assay. The GSH is a copper chelator that reduces the sensitivity of cells to copper and can be used as an inhibitor of cuproptosis.

### 2.15 Transfection of siRNA

The siRNA and Lipofectamine RNAiMAX were diluted using reduced serum medium at the recommended concentrations in the instruction. After that, the two diluted solutions were mixed and stood for 20 min at room temperature. We then added the mixture to the culture dish containing cells and continued to incubate the cells for 48 h.

### 2.16 CCK-8 cell proliferation assay

We cultured the HCAECs in 96-well plates. When the cell density of each well reached approximately 80%, CuCl2 with the following different concentrations were added to each well for 24 h: 5 μM, 10 μM, 25 μM, 50 μM, 75 μM and 100 μM. After that, we added 10 μL CCK8 solution to each well. In the end, we measured the absorbance at 450 nm after 2 h.

### 2.17 Western blot

The proteins extracted from HCAECs were separated by electrophoresis. After that, the proteins were transferred to PVDF membrane, blocked with 5 % bovine serum albumin for 1 h, and incubated with FDX1, LIAS and HSP70 primary antibody at 4 °C overnight. Next, we incubated the corresponding secondary antibodies at room temperature for 1 h. In the end, the chemiluminescence reaction and protein blot imaging were conducted.

### 2.18 RT-qPCR

We set the reverse transcription process as follows: 25 °C for 5 min, 55 °C for 15 min, 85 °C for 5 min. We set the amplification process as follows: pre-denaturation for 5 min at 95 °C, followed by 40 cycles of 95 °C for 10 s and 60 °C for 30 s. We employed the △△Ct method to evaluate the expression level of mRNA. The Supplementary Table S5 presented the primer sequences (synthesis of primer was performed by Generay Biotech).

### 2.19 Statistical analysis

We used the R 4.2.1 software to conduct bioinformatics analysis and employed the GraphPad Prism 7.0 software to conduct data analysis in vitro experiment. One-way ANOVA was conducted to compare three or more groups of data. The *P* value less than 0.05 (*P* < 0.05) was considered statistically significant.

## 3 Results

### 3.1 Identification of A-CRGs

**Fig. 1:**
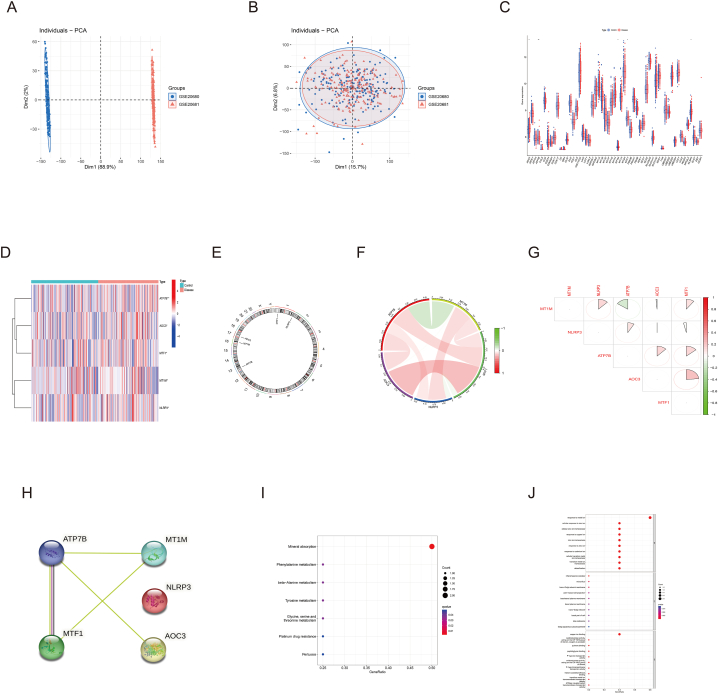
Differential analysis and functional analysis of A-CRGs. (A) PCA analysis before batch correction. (B) PCA analysis after batch correction. (C) Box plot of CRGs. (D) Heatmap of 5 A-CRGs. (E) The position of 5 A-CRGs on the chromosome. (F–G) Correlation analysis of 5 A-CRGs. (H) PPI network diagram of 5 A-CRGs. (I–J) GO analysis and KEGG analysis of 5 A-CRGs.

The GSE20680 and GSE20681 datasets showed two distinct clusters before the removal of batch effect and clustered together after batch correction (Fig. 1A and B). We found 59 CRGs based on the published literature. Then, differential expression analysis of these CRGs between atherosclerosis and control group in the training set was performed. The result showed that a total of 5 A-CRGs were identified and all of them were up-regulated in the disease group (Fig. 1C and D). The chromosome positions of 5 A-CRGs were presented in Fig. 1E. Next, the correlation analysis and PPI network analysis were conducted on the 5 A-CRGs, and the results showed that there is some correlation and interaction between them (Fig. 1F–H). Finally, the GO analysis and KEGG analysis were conducted on the 5 A-CRGs, and the results manifested that they were related to the biological processes of cuproptosis, such as alanine metabolism, copper ion reaction, and inflammation (Fig. 1I and J). The names of the proteins encoded by these 5 A-CRGs and their roles in cuproptosis were summarized in Supplementary Table S6.

### 3.2 Functional analysis of the A-CRGs

**Fig. 2:**
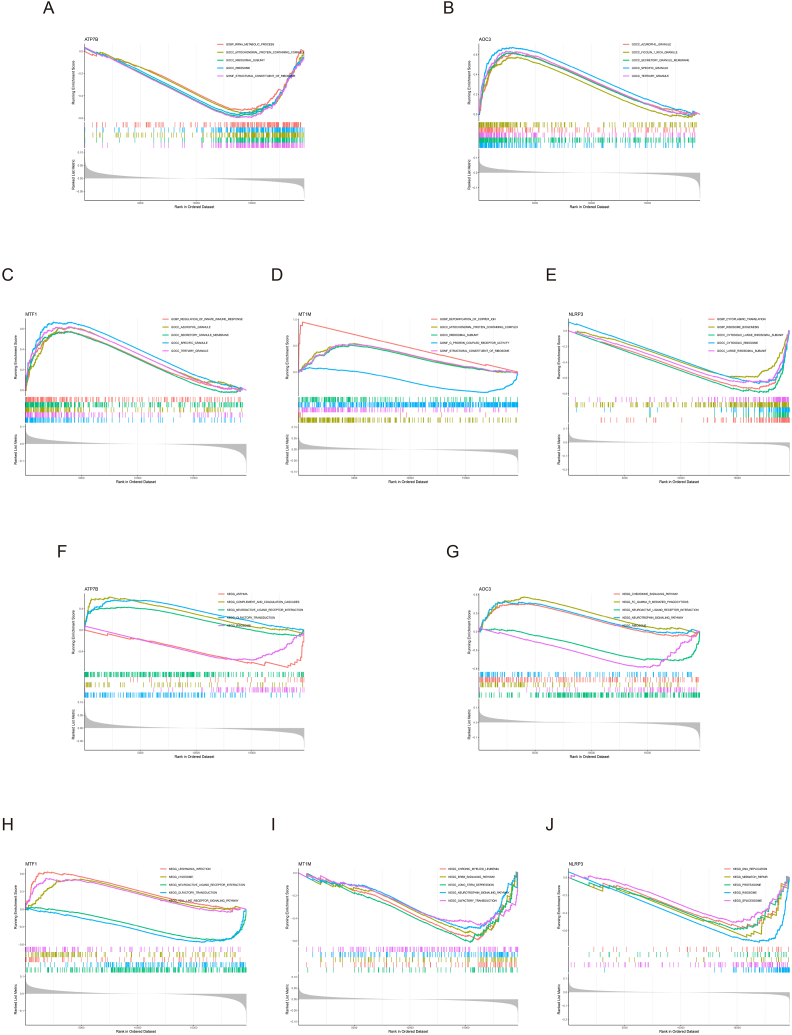
Single-gene GSEA of A-CRGs. (A–E) GSEA-GO analysis of 5 A-CRGs. (F–J) GSEA-KEGG analysis of 5 A-CRGs.

**Fig. 3:**
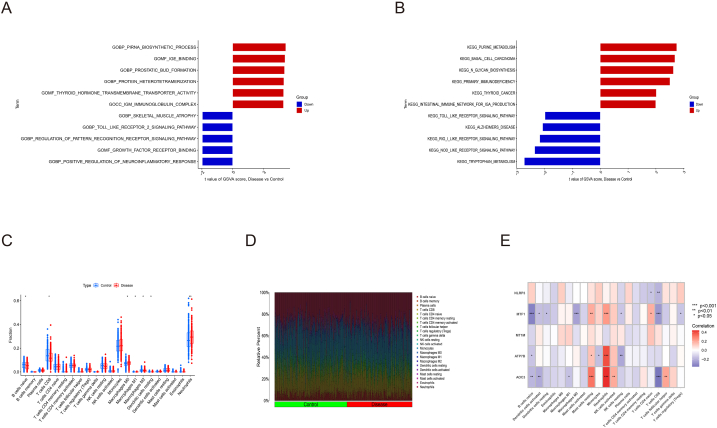
Differential function analysis between atherosclerosis and control group. (A) GSVA-GO analysis of atherosclerosis and control group. (B) GSVA-KEGG of atherosclerosis and control group. (C) Box plot of immune cells infiltration. (D) Bar plot of immune cells infiltration. (E) Correlation analysis of A-CRGs and immune cells.

We conducted the single-gene GSEA to investigate the biological processes which regulated by the 5 A-CRGs. The results manifested that the A-CRGs mainly regulated the biological processes such as mitochondrial proteins containing complex, innate immune response, copper ion detoxification, chemokine and toll like receptor signaling pathway (Fig. 2A–J). After that, we conducted GSVA between atherosclerosis and control group. The results manifested that biological processes such as immunoglobulin complex, primary immunodeficiency signaling pathway were significantly activated in the atherosclerosis group (Fig. 3A and B). Due to the fact that the occurrence of atherosclerosis is closely related to immune inflammatory response and the GSVA results also indicated that the immune response was activated in the atherosclerosis group, we analyzed the difference of immune cell infiltration between atherosclerosis and control group. The results demonstrated that the infiltration levels of M1 type macrophages and neutrophils were up-regulated in the atherosclerosis group, while the infiltration levels of M0 type and M2 type macrophages were down-regulated in the atherosclerosis group. This finding further demonstrated that the occurrence of atherosclerosis was associated with immune response (Fig. 3C and D). The GO and KEGG analysis as well as GSEA of the 5 A-CRGs showed that they were related to immune inflammatory response. Thus, we further analyzed the association between 5 A-CRGs and immune cells, and found that 4 genes were correlated with the immune cells (Fig. 3E).

### 3.3 Construction of diagnostic model on the basis of A-CRGs

**Fig. 4:**
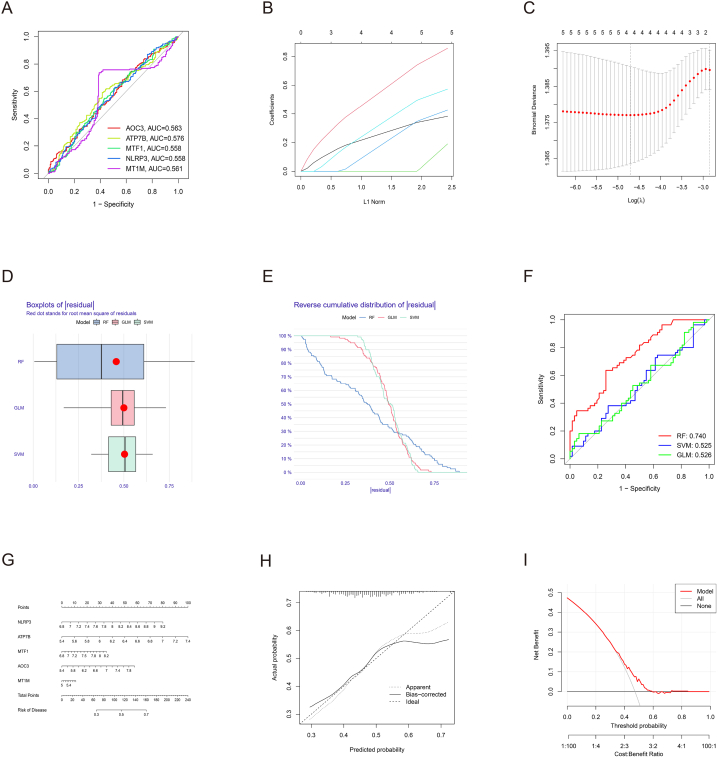
Assessment of the value of 5 A-CRGs in atherosclerosis diagnosis. (A) Evaluation of the diagnostic value of 5 A-CRGs individually by ROC analysis. (B) LASSO coefficient of 5 A-CRGs. (C) Optimal lambda value of LASSO regression. (D–F) Residual boxplot, cumulative residual distribution curve and ROC curve of machine learning algorithm. (G) Nomogram of 5 A-CRGs. (H–I) Calibration curve and DCA of the nomogram.

First, the diagnostic performance of the 5 A-CRGs on atherosclerosis was assessed respectively, and we found that all the individual genes showed poor diagnostic performance (Fig. 4A). Then, we attempted to enhance the diagnostic performance via establishing multi-gene association diagnostic models. The result of LASSO regression showed that 4 key A-CRGs had the best diagnostic value (AOC3, ATP7B, MTF1, NLRP3) (Fig. 4B and C). After that, 3 machine learning models (RF, SVM and GLM) were established on the basis of 4 key A-CRGs. We used three methods, i.e., residual boxplots, cumulative residual distribution curves and ROC curves, to select the best model. Due to the small number of key A-CRGs, and the number of key A-CRGs screened by LASSO regression is at the critical value of 4 and 5, we built the machine learning models based on 4 key A-CRGs and 5 A-CRGs respectively. The results demonstrated that the RF model had the best diagnostic performance regardless of whether the model was constructed based on 4 genes or 5 genes, and the diagnostic efficiency of the 4-gene model and the 5-gene model was almost the same (area under ROC curve was 0.764 and 0.740 respectively). Therefore, we still selected 5 A-CRGs as the key genes for subsequent molecular subtype studies. The results of machine learning model construction for 4-gene and 5-gene were shown in Supplementary Figs. S1A–C and Fig. 4D–F respectively. In the end, the nomogram was established based on 4 A-CRGs and 5 A-CRGs respectively to predict the risk of atherosclerosis. The results manifested that the prediction efficacy of the nomogram was well (Supplementary Figs. S1D–F and Fig. 4G–I).

### 3.4 Identification of cuproptosis-related molecular subtype for atherosclerosis

**Fig. 5:**
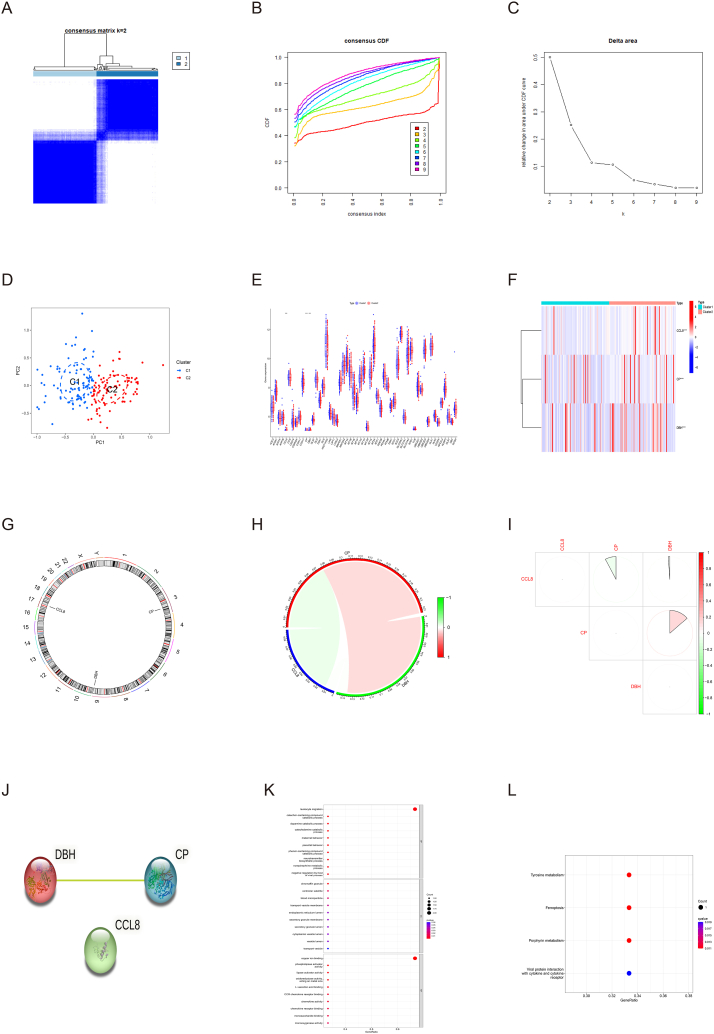
Identification of cuproptosis-related molecular subtypes clusters and functional analysis of M-CRGs. (A) Consensus clustering matrix. (B) CDF curves. (C) CDF delta area curves. (D) PCA of 2 molecular subtypes clusters. (E) Box plot of CRGs. (F) Heatmap of 3 M-CRGs. (G) The position of 3 M-CRGs on the chromosome. (H–I) The correlation analysis of 3 M-CRGs. (J) The PPI network diagram of 3 M-CRGs. (K) GO analysis and KEGG analysis of 3 M-CRGs.

We conducted the unsupervised clustering analysis for atherosclerosis group on the basis of 5 A-CRGs to discover the cuproptosis-related molecular subtype of atherosclerosis. The results demonstrated that when the value of k was 2, the numbers of clusters were the most appropriate (Fig. 5A–C, Supplementary Fig. S2A-G and S3A-I). Thus, we divided the atherosclerosis group into 2 cuproptosis-related molecular subtypes clusters (cluster 1 and 2). The results of PCA manifested that these 2 clusters were clearly separated (Fig. 5D).

### 3.5 Identification of M-CRGs

First, we conducted the differential expression analysis of CRGs between the cluster 1 and 2. The result showed that a total of 3 M-CRGs were identified and all of them were up-regulated in the cluster 2 (Fig. 5E and F). The chromosome positions of the 3 M-CRGs were presented in Fig. 5G. Next, we conducted the correlation analysis and PPI network analysis based on the 3 M-CRGs, and the results demonstrated that there was some association and interaction between them (Fig. 5H–J). Finally, we conducted the GO and KEGG analysis based on the 3 M-CRGs, and the results manifested that the 3 M-CRGs were involved in the processes related to cuproptosis, such as copper ion binding, chemokine activation, and leukocyte migration (Fig. 5K and L). The names of the proteins encoded by these 3 M-CRGs and their roles in cuproptosis were summarized in Supplementary Table S7.

### 3.6 Functional analysis of the M-CRGs

**Fig. 6:**
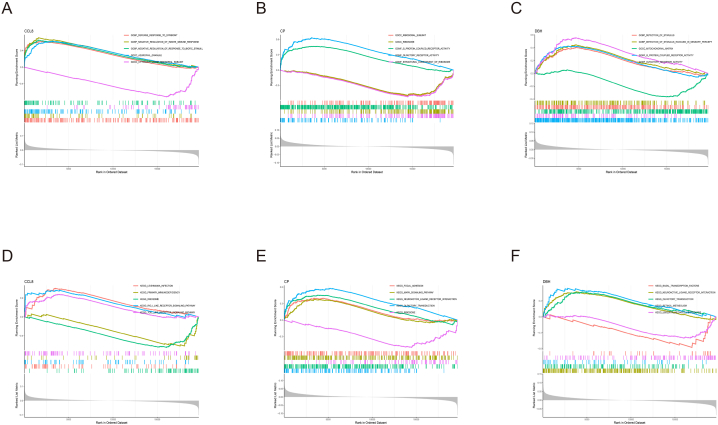
Single-gene GSEA of M-CRGs. (A–C) GSEA-GO analysis of 3 M-CRGs. (D–F) GSEA-KEGG analysis of 3 M-CRGs.

**Fig. 7:**
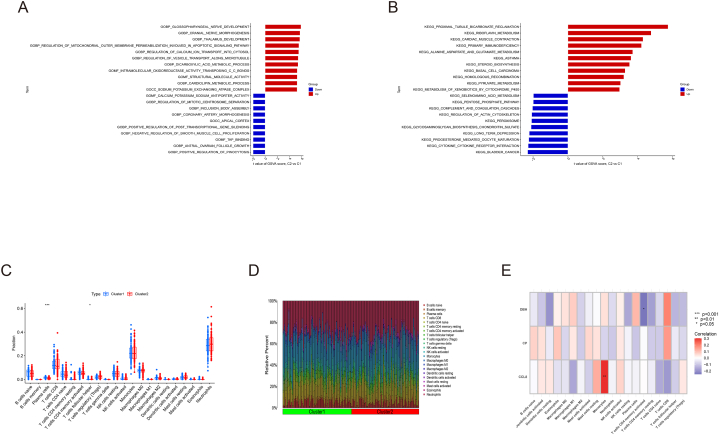
Differential function analysis between 2 molecular subtypes clusters. (A) GSVA-GO analysis of 2 molecular subtypes clusters. (B) GSVA-KEGG analysis of 2 molecular subtypes clusters. (C) Box plot of immune cells infiltration. (D) Bar plot of immune cells infiltration. (E) Correlation analysis of M-CRGs and immune cells.

We conducted the single-gene GSEA to investigate the biological processes which regulated by the 3 M-CRGs. The results manifested that the M-CRGs mainly regulated the biological processes such as mitochondrial function, primary immunodeficiency, toll like receptor and MAPK signaling pathway (Fig. 6A–F). After that, we conducted GSVA between the cluster 1 and 2, and we found that biological functions such as coronary artery morphogenesis were significantly activated in the cluster 1. The biological functions such as mitochondrial outer membrane permeabilization, smooth muscle cell proliferation, primary immunodeficiency and pyruvate metabolism were significantly activated in the cluster 2 (Fig. 7A and B). Since GSEA results suggested that these M-CRGs could regulate immunoinflammatory responses, and GSVA results also showed that there were differences in the activation degree of immunoinflammatory related signaling pathways between the two clusters, we analyzed the difference of immune cell infiltration between the 2 clusters. We found that the infiltration levels of T cells follicular helper and plasma cells were up-regulated in the cluster 1, further suggesting that the 2 molecular subtype clusters showed differences in immune environment (Fig. 7C and D). The GO analysis and KEGG analysis as well as GSEA of the 3 M-CRGs indicated that they were related to immune inflammatory response. Hence, we analyzed the association between 3 M-CRGs and immune cells, and found that 2 genes were associated with the immune cells (Fig. 7E).

### 3.7 Construction of the model for identification of cuproptosis-related molecular subtypes based on M-CRGs

**Fig. 8:**
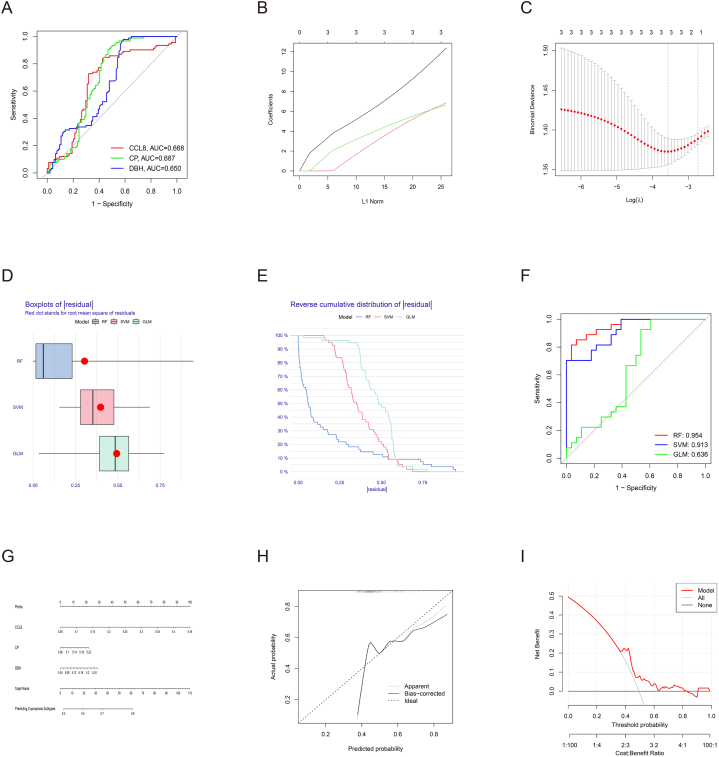
Assessment of the value of 3 M-CRGs in molecular subtype identification. (A) Assessment of the identification value of 3 M-CRGs via ROC analysis. (B) LASSO coefficient of 3 M-CRGs. (C) Optimal lambda value of LASSO regression. (D–F) Residual boxplot, cumulative residual distribution curve and ROC curve of machine learning algorithm. (G) Nomogram of 3 M-CRGs. (H–I) Calibration curve and DCA of the nomogram.

First, the molecular subtypes identification performance of the 3 M-CRGs on atherosclerosis was assessed respectively, and the results manifested that all genes showed poor identification performance (Fig. 8A). Then, we attempted to improve the identification performance via establishing multi-gene association models. The result of LASSO regression shown that all the 3 M-CRGs had the best diagnostic value (CCL8, CP, DBH) (Fig. 8B and C). After that, 3 machine learning models (RF, SVM and GLM) were established on the basis of 3 M-CRGs. We used three methods, i.e., residual boxplots, cumulative residual distribution curves and ROC curves, to select the best identification model. We found that the RF model had the best identification efficiency (Fig. 8D–F). In the end, the nomogram was established based on 3 M-CRGs to predict the molecular subtypes more accurately. The results indicated well prediction efficacy of the nomogram (Fig. 8G–I).

### 3.8 Verification of the efficacy of the diagnostic model

**Fig. 9:**
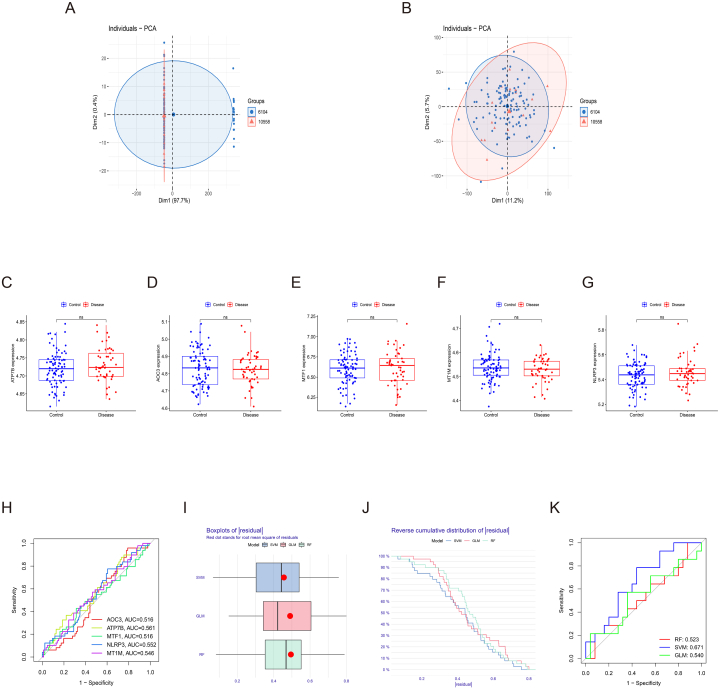
Validation of the value of 5 A-CRGs in atherosclerosis diagnosis. (A) PCA analysis before batch correction. (B) PCA analysis after batch correction. (C–G) Box plot of 5 A-CRGs. (H) Assessment of the diagnostic value of 3 M-CRGs via ROC analysis. (I–K) Residual boxplot, cumulative residual distribution curve and ROC curve of machine learning algorithm.

The GSE20129 dataset consists of 2 platform files (6104 and 10558). The platform files 6104 and 10558 showed 2 distinct clusters before the removal of batch effect and clustered together after batch correction (Fig. 9A and B). After that, the levels of 5 A-CRGs were verified in validation dataset. The results demonstrated that the levels of 3 A-CRGs (ATP7B, MTF1, NLRP3) in validation dataset were in line with those in training dataset, although they were not significantly differentially expressed between the control and disease groups (Fig. 9C–G). Then, the diagnostic performance of 5 A-CRGs in validation dataset was assessed respectively, and we found that all genes showed poor diagnostic performance (Fig. 9H). Next, the machine learning models were established on the basis of 5 A-CRGs in the validation set. We found that SVM yielded better diagnostic performance than the single gene (Fig. 9I–K). Finally, the nomogram was established based on 5 A-CRGs to validate the diagnostic performance of these genes and the result was illustrated in the Supplementary Figs. S4A–C.

### 3.9 Verification of the efficacy of the molecular subtypes identification model

**Fig. 10:**
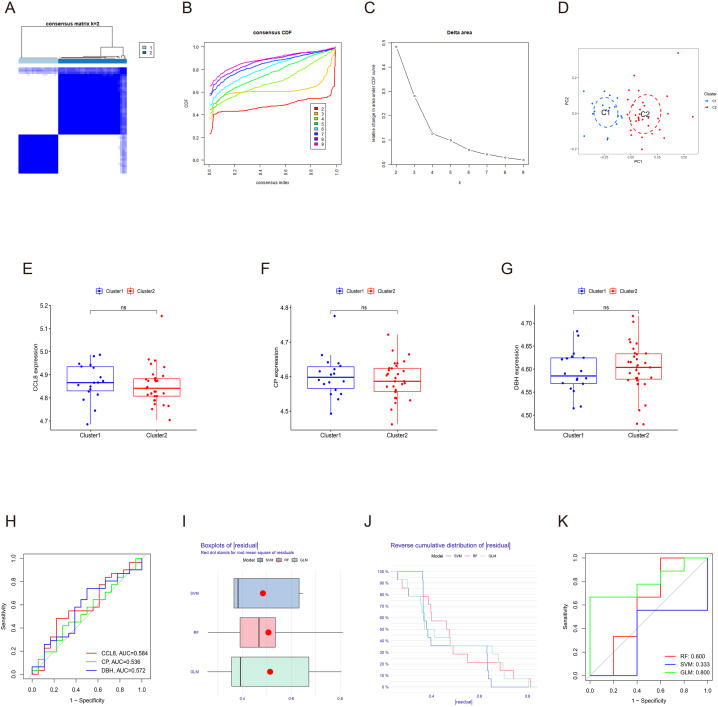
Validation of the value of 3 M-CRGs in molecular subtype identification. (A) Consensus clustering matrix. (B) CDF curves. (C) CDF delta area curves. (D) PCA of 2 molecular subtypes clusters. (E–G) Box plot of 3 M-CRGs. (H) Assessment of the identification value of 3 M-CRGs via ROC analysis. (I–K) Residual boxplot, cumulative residual distribution curve and ROC curve of machine learning algorithm.

The unsupervised cluster analysis of atherosclerosis group was conducted in the validation set based on the 5 A-CRGs. The results demonstrated that when the value of k was 2, the number of clusters was the most appropriate (Fig. 10A–C, Supplementary Fig. S5A-G and S6A-I). The results of PCA manifested that the 2 clusters were markedly separated (Fig. 10D). After that, the levels of 3 M-CRGs were validated in validation dataset. The results demonstrated that the levels of CCL8 gene and CP gene were up-regulated in cluster 1, the expression level of DBH gene was down-regulated in cluster 1, although there was no statistical difference (Fig. 10E–G). Next, we evaluated the identification value of the 3 M-CRGs in the validation dataset respectively. We found that all genes showed poor identification performance (Fig. 10H). Subsequently, the machine learning models were established on the basis of 3 M-CRGs in validation dataset and we found that GLM showed better identification performance than the single gene (Fig. 10I–K). Finally, the nomogram was established based on 3 M-CRGs to validate the identification performance of these genes. The establishment and evaluation results of the nomogram were shown in the Supplementary Figs. S7A–C.

### 3.10 Effects of CuCl_2_ on the cell viability, expression levels of 5 A-CRGs and immunoinflammatory factors in HCAECs

**Fig. 11:**
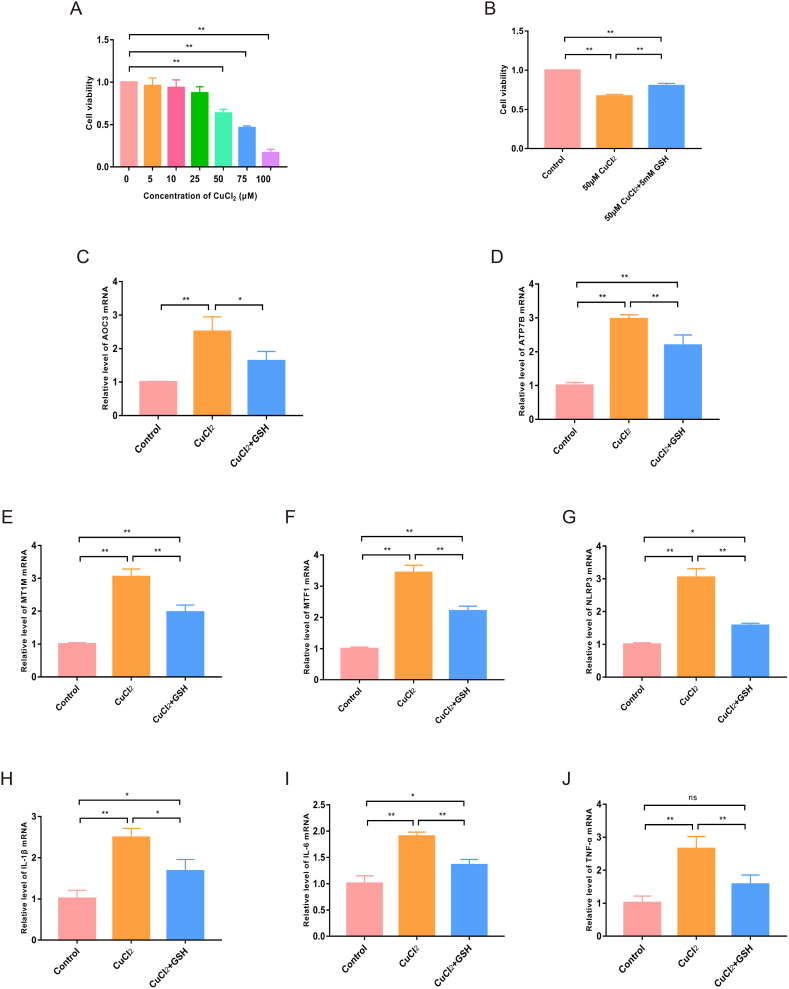
Effects of CuCl_2_ on the cell viability, expression levels of 5 A-CRGs and immunoinflammatory factors in HCAECs. (A) The effects of CuCl_2_ on HCAECs viability. (B) The effects of GSH on HCAECs viability. (C–G) The mRNA levels of 5 A-CRGs in HCAECs were tested by RT-qPCR. (H–J) The mRNA expression levels of IL-1β, IL-6 and TNF-α in HCAECs were tested by RT-qPCR. (*P < 0.05, **P < 0.01).

Next, we attempted to verify the role of cuproptosis in atherosclerosis and changes in levels of 5 A-CRGs through in vitro experiments. As we all know, there is no direct in vitro model that can represent atherosclerosis. The in vitro model generally needs to be selected according to the specific cellular mechanism involved in the study. Commonly used cell models include endothelial cell model, macrophage model, and smooth muscle cell model [19]. Endothelial cell dysfunction is the initial event of atherosclerosis and one of the most critical links in the development of atherosclerosis [20]. Previous studies have shown that programmed cell death mechanisms (ferroptosis, apoptosis, pyroptosis and autophagy) affect atherosclerotic progression primarily through impaction of endothelial cell function [21]. These studies typically determine whether a certain cell death pattern affects atherosclerosis by the following methods [22,23]: Specific cell death inducers or inhibitors were used to treat vascular endothelial cells, such as ferroptosis inducer Erastin and ferroptosis inhibitor ferrostatin-1. If Erastin treatment could reduce the vascular endothelial cell viability and change the expression levels of ferroptosis marker proteins, and meanwhile, ferrostatin-1 combined with Erastin treatment could reduce the influence of Erastin on cell viability and marker proteins expression levels, it indicates that ferroptosis can accelerate atherosclerosis by damaging endothelial cell function. Therefore, following previous research, we treated HCAECs with cuproptosis inducer CuCI_2_ and inhibitor GSH, and observed cell viability and changes in levels of cuproptosis marker proteins HSP70, LIAS and FDX1, so as to verify whether cuproptosis affects atherosclerosis by regulating endothelial cell function. In addition, in the case of cuproptosis in endothelial cells, the levels of 5 A-CRGs were detected to verify whether the levels of A-CRGs could reflect the degree of cuproptosis in endothelial cells. First, we screened the appropriate concentration of CuCl_2_ to construct the cuproptosis model of HCAECs by CCK-8 experiment. The results showed that CuCl_2_ could induce significant cell death when the concentration reached 50 μM (Fig. 11A). Thus, 50 μM concentration of CuCl_2_ was selected for subsequent experiments. To validate the role of copper in endothelial cell death, HCAECs were treated with GSH combined with CuCl_2_. The results showed that cell death was significantly inhibited when the GSH was used to bind copper, further suggesting that copper could induce endothelial cell death and thus lead to the development of atherosclerosis (Fig. 11B). Then, we detected the levels of 5 A-CRGs in endothelial cell of CuCl_2_ treatment group. The results demonstrated that the levels of 5 A-CRGs in the CuCl_2_ group were up-regulated, while these trends were significantly reversed in the GSH combined with CuCl_2_ group (Fig. 11C–G). These results indicated that 5 A-CRGs could reflect the degree of cuproptosis in endothelial cells. The higher the expression levels of these genes, the more serious the degree of cuproptosis in endothelial cells. Considering that the levels of these 5 A-CRGs in peripheral blood of atherosclerosis patients were also significantly up-regulated, it further suggested that endothelial cell cuproptosis might be related to the pathogenesis of atherosclerosis. In addition, since the results of bioinformatics analysis in this study suggested that cuproptosis was related to immune inflammatory response, and other previous studies also found a certain correlation between cuproptosis and immune inflammation [24,25], we further explored whether cuproptosis in endothelial cells was accompanied by an intensification of immune inflammatory response. We detected the mRNA levels of immunoinflammatory factors in HCAECs, and found that the levels of immunoinflammatory factors were up-regulated in CuCl_2_ treated cells, while GSH treatment could weaken these changes (Fig. 11H–J). These results suggested that cuproptosis of endothelial cells is accompanied by the intensification of immune inflammatory response.

### 3.11 Effects of 5 A-CRGs on cuproptosis and expression levels of immunoinflammatory factors in HCAECs

**Fig. 12:**
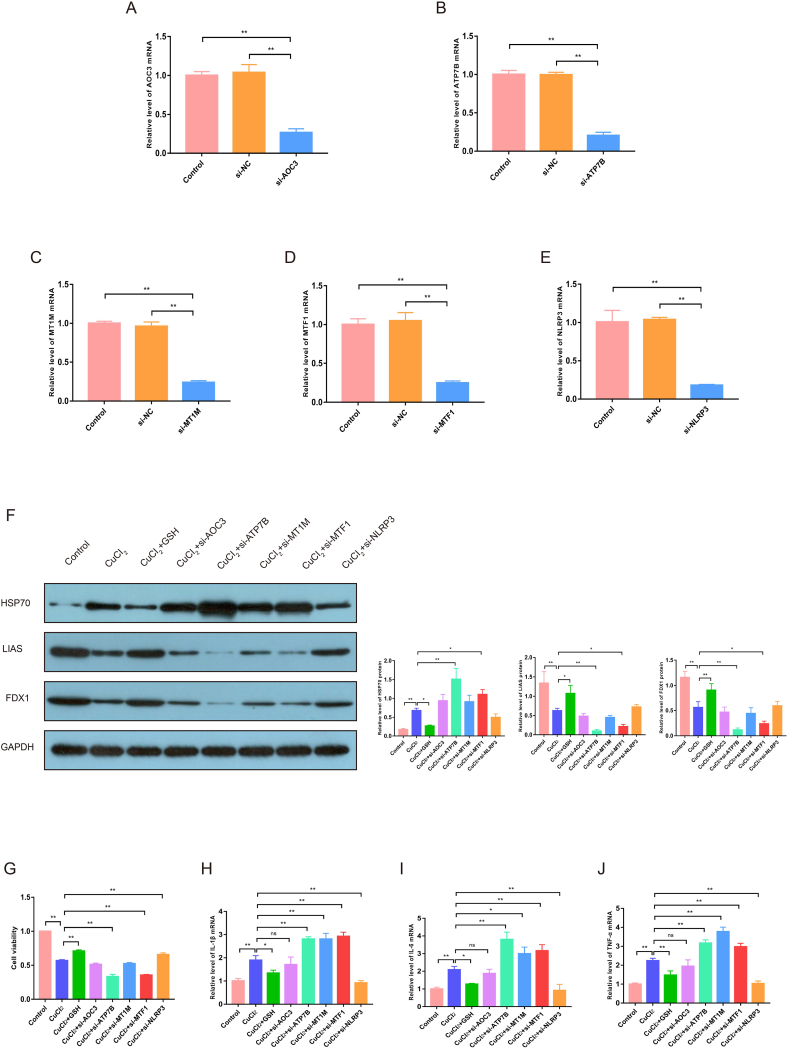
Effects of 5 A-CRGs on cuproptosis and expression levels of immunoinflammatory factors in HCAECs. (A–E) The mRNA levels were tested by RT-qPCR after knockdown of 5 A-CRGs. (F) The protein levels of HSP70, LIAS, and FDX1 in HCAECs were tested by Western blot (n = 3). (G) The effects of 5 A-CRGs knockdown on HCAECs viability. (H–J) The mRNA levels of IL-1β, IL-6 and TNF-α in HCAECs were tested by RT-qPCR after knockdown of 5 A-CRGs. (*P < 0.05, **P < 0.01).

To further investigate the role of 5 A-CRGs in cuproptosis of endothelial cells, we performed gene knockdown verification experiments. First, we used siRNA to knockdown these 5 genes in HCAECs. The results manifested that the mRNA levels of target genes in the siRNA groups were down-regulated and the levels in the si-NC groups had no significant changes (Fig. 12A–E). Then, we detected the expression changes of cuproptosis marker proteins in cells after genes knockdown to reflect the severity of cuproptosis. The results showed that the severity of cuproptosis was further aggravated after ATP7B and MTF1 genes knockdown (protein levels of FDX1 and LIAS were down-regulated, and protein levels of HSP70 were up-regulated). However, there were no significant changes in the severity of cuproptosis after knockdown of AOC3, MT1M and NLRP3 genes (Fig. 12F and Supplementary Figs. S8A–D). These results indicated that ATP7B and MTF1 genes may directly affect the degree of cuproptosis, while AOC3, MT1M and NLRP3 genes only change with the degree of cuproptosis, but do not directly affect the cuproptosis. Next, we examined the changes in cell viability after genes knockdown. The results manifested that the viability of cells decreased after knockdown of ATP7B and MTF1 genes, the viability of cells increased after knockdown of NLRP3 gene, while the viability of cells did not change significantly after knockdown of AOC3 and MT1M genes (Fig. 12G). These results further suggested that ATP7B and MTF1 genes may directly affect the degree of cuproptosis in cells and thus reduce the viability of cells. In addition, NLRP3 gene knockdown affected the cell viability but did not affect the degree of cuproptosis, which we believe may be because the NLRP3 gene knockdown could affect the pyroptosis. Finally, since the results of bioinformatics analysis in this study suggested that A-CRGs were related to immunoinflammatory response, we further explored whether the levels of immunoinflammatory factors in endothelial cells would change after the knockdown of these 5 A-CRGs. We found that the mRNA levels of immunoinflammatory factors in HCAECs were up-regulated after the knockdown of ATP7B, MTF1 and MT1M, and down-regulated after the knockdown of NLRP3, while the levels of immunoinflammatory factors were not significantly changed after the knockdown of AOC3 (Fig. 12H–J). These results preliminarily indicated that some A-CRGs are not only associated with cuproptosis, but also with immune inflammatory response, further suggesting that there was some association between cuproptosis and immune inflammatory response. However, the exact mechanism still needs to be validated via further investigation.

### 3.12 Regulatory molecular prediction of A-CRGs and M-CRGs

**Fig. 13:**
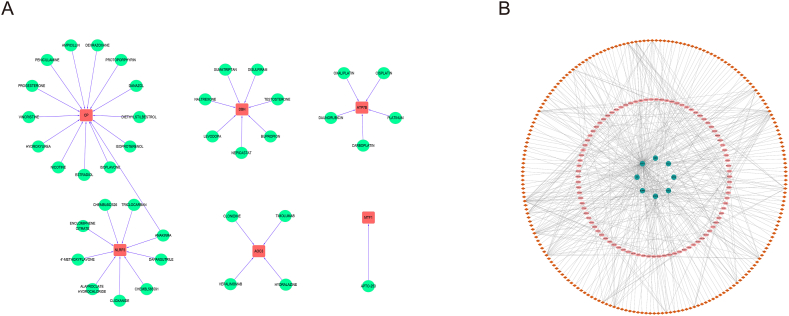
Prediction of key genes targeted drugs and ceRNA regulatory network (5 A-CRGs and 3 M-CRGs). (A) Targeted drugs regulatory network. (B) ceRNA regulatory network.

As we all know, the function of genes is mainly achieved by transcription into mRNA, which is then translated into proteins. The DGIdb database contains a large amount of information on the interactions of compounds and proteins [26]. Compounds in the database that can interact with a particular protein are often considered as potential targeted regulatory drugs for that protein. The miRNA can bind target mRNA in the form of complementary base pairing, regulate mRNA translation process, and then affect protein synthesis [27]. The lncRNA can competitively binds to target miRNA in the form of complementary base pairing [28]. Once miRNA bind to lncRNA, it can no longer bind to mRNA. Therefore, lncRNA can regulate the binding process of miRNA and mRNA, and then affect the translation process of mRNA, and finally affect protein synthesis. This regulatory mode is called the ceRNA regulatory network [29]. As we have confirmed in vitro experiments that some CRGs can affect endothelial cell viability and cuproptosis degree, and they were promising therapeutic targets for atherosclerosis. Therefore, the exploration of substances that can regulate these key CRGs will help to find new therapies for atherosclerosis and has important clinical significance. The lncRNA, miRNA and compounds can regulate gene function at mRNA and protein levels respectively. Exploring lncRNA, miRNA and compounds that can regulate the function of key CRGs might provide significant insights into the treatment of atherosclerosis, that is, regulating endothelial cell cuproptosis to alleviate atherosclerosis. In this study, drugs that might target binding of the key CRGs was predicted using the DGIdb database. The predicted results showed that total of 50 drugs were obtained. Among these drugs, 14 drugs might target binding of the CP, 9 drugs might target binding of the NLRP3, 7 drugs might target binding of the DBH, 5 drugs might target binding of the ATP7B, 4 drugs might target binding of the AOC3 and 1 drug might target binding of the MTF1 (Fig. 13A). However, no drugs were found for MT1M and CCL8. Then, the ceRNA network was established based on 5 A-CRGs and 3 M-CRGs. The network consisted of 302 nodes (8 CRGs mRNA, 203 miRNAs and 91 lncRNAs) and 411 edges (Fig. 13B). The Supplementary Table S8 illustrated the detailed interactive relationships of all mRNA, miRNA and lncRNA in this network.

## 4 Discussion

Copper is an important helper factor of transporters and enzymes in vivo. It not only regulates the redox balance of cells, but also mediates a specific cell death mode, that is, cuproptosis. Previous studies have reported that increasing bioavailable copper could accelerate the progression of atherosclerosis, but the specific reason is not clear, and cuproptosis provides a possibility to elucidate this mechanism [30]. Although cuproptosis is a novel cell death pattern discovered recently, several studies have reported its association with a variety of neoplastic and non-neoplastic diseases [31,32]. To our knowledge, this study is the first to find the potential correlation between cuproptosis and atherosclerosis.

In this study, 2 GEO datasets were used to identify 5 CRGs with significant expression differences between control group and atherosclerosis group. After that, we analyzed the interaction between these 5 key CRGs, as well as the biological processes involved in the regulation of these genes. The results indicated that there was an obvious interaction between them. The biological processes involved in them were mainly copper ion metabolism, followed by immune inflammatory response. Subsequently, we constructed diagnostic models of atherosclerosis based on these genes and verified the efficacy of the models. The results indicated that these genes had satisfactory combined diagnostic value. On the other hand, based on these 5 genes, we identified 2 clusters of atherosclerosis molecular subtypes associated with cuproptosis. The functional analysis results manifested that there were many differences in biological functions and signaling pathways between the 2 clusters, such as coronary artery morphogenesis, mitochondrial outer membrane permeabilization, primary immunodeficiency and so on. In addition, we also identified 3 M-CRGs and constructed molecular subtype identification models based on these 3 M-CRGs. The results indicated that these genes had well molecular subtype discrimination value. Finally, we verified the efficacy of the key genes for diagnosis and molecular subtypes identification of atherosclerosis in another GEO dataset. In addition, the targeted drugs and ceRNA network of the A-CRGs and M-CRG were obtained from the online databases, which will be conducive to the research of relevant therapeutic targets and drugs in the future. These results not only revealed the potential association between cuproptosis and atherosclerosis, but also provided some evidence for the application of CRGs in the diagnosis and molecular subtypes identification of atherosclerosis. Further research on cuproptosis and atherosclerosis in the future is expected to elucidate the pathogenesis of atherosclerosis, discover novel diagnostic biomarkers and explore more scientific and effective individualized treatment methods. It should be noted that the research samples of the 3 GEO datasets selected in this study were all human peripheral blood samples (whole blood samples) rather than arterial tissue samples. In general, in studying the role of cuproptosis in atherosclerotic pathophysiology, the best sample is human artery tissue. However, in actual clinical situations, human artery tissue is difficult to obtain. In addition, when it comes to further investigating the value of CRGs in the diagnosis of atherosclerosis, it is obviously unreasonable to use arterial tissue, because obtaining arterial tissue can be very traumatic to the human body and difficult to apply in routine diagnosis. Compared with arterial tissue, the process of obtaining peripheral blood samples is simple and non-invasive. Relevant studies have found that peripheral blood cells not only contribute to the auxiliary diagnosis of atherosclerosis, but also indirectly reflect the degree of programmed death in tissue cells (autophagy and cuproptosis) [33]. One study found that the levels of autophagy markers in peripheral blood cells can reflect the degree of interaction between vascular endothelial cells and white blood cells, the degree of activation of autophagy related pathways, and the risk of atherosclerosis [34]. Another study demonstrated an association between levels of CRGs in tumor tissues and levels of CRGs in peripheral blood, suggesting that the levels of CRGs in peripheral blood cells could reflect the degree of cuproptosis in tissues to a certain extent [35]. Moreover, during the occurrence of atherosclerosis, peripheral blood cells (monocytes, lymphocytes, neutrophils) are recruited into vascular endothelial cells and subintimal cells under the action of various chemokines and adhesion molecules [36,37], which may regulate the degree of cuproptosis in vascular endothelial cells and subintimal cells and affect the progression of atherosclerosis. Therefore, peripheral blood cells could not only reflect the degree of cuproptosis in vascular cells, but also might regulate the cuproptosis in vascular cells. Hence, they are suitable sample for investigating the role of cuproptosis in pathophysiology of atherosclerosis and the value of CRGs in the diagnosis of atherosclerosis.

A total of 5 key atherosclerosis diagnostic genes were identified in this study, and the bioinformatics analysis results demonstrated that all these genes were up-regulated in the atherosclerosis group. ATP7B is a copper transporting ATPase, belonging to the family of P-type ATPases. It is one of the most important molecules in regulating the content of copper in cells. When ATP7B gene expression level is reduced, intracellular copper excretion is reduced, and related biological processes mediated by copper are activated [38]. Correspondingly, ATP7B expression levels will be upregulated when copper is used to stimulate cells in vitro to promote the excretion of excess copper from the cells [39]. Tsvetkov et al. have clearly reported that ATP7B gene knockout could promote the occurrence of cuproptosis, but no studies have reported the association between ATP7B and atherosclerosis [7]. MTF1 is a metal regulatory transcription factor, which could transfer from cytoplasm to nucleus when copper levels rise and play a role in maintaining the homeostasis of copper [40]. Previous research has shown that copper could induce the up-regulation of MTF1 expression in endothelial cells [41]. In addition, another study has reported that MTF1 could positively regulate the expression level of ATP7B [42]. All these phenomena suggested that MTF1 might regulate copper-mediated cuproptosis of endothelial cells, and may also influence the occurrence and development of atherosclerosis through this mechanism. NOD-like receptor thermal protein domain associated protein 3 (NLRP3) is an important member of the NOD-like receptor family, and it is one of the important molecules in regulating pyroptosis [43]. In recent years, it has been found that the level of NLRP3 is positively associated with that of ATP7A [44]. In addition, copper treatment increased NLRP3 expression, and NLRP3 knockdown mitigate neurodegeneration and cognitive decline in ATP7B knockout mice [45]. These findings suggested that NLRP3 may have some correlation with cuproptosis. Amine oxidase copper containing 3 (AOC3) is a member of semicarbazide-sensitive amine oxidase family. It was found that the level of AOC3 was positively correlated with copper content [46], serum AOC3 concentration could predict the risk of atherosclerosis [47], and AOC3 expression level in arterial tissue was also positively correlated with the severity of atherosclerosis [48]. MT1M is a member of the metallothioneins protein family, which could bind copper, zinc and other heavy metals and regulate the homeostasis of copper [49]. Previous research has shown that copper could induce the up-regulation of MT1M expression in endothelial cells, and knocking down some subtypes of metallothioneins protein family could inhibit the proliferation of endothelial cells [50]. These results suggested a potential relationship between copper and MT1M as well as atherosclerosis and MT1M. In addition, another study has found that when the epithelial cells were treated with high concentration of copper, not only the MT1M level, but also the heat shock protein family level was found to be increased [51]. The heat shock protein is one of the signature proteins in the occurrence of cuproptosis, which further suggests a potential link between MT1M and cuproptosis [7].

To further verify the role of the 5 genes in atherosclerosis and cuproptosis, we conducted in vitro experiments. The treatment of HCAECs with CuCl_2_ resulted in increased expression of intracellular cuproptosis marker protein and decreased cell viability, indicating that the endothelial cell cuproptosis model have been constructed successfully. Typically, endothelial cell death or dysfunction is the initial event in the development of atherosclerosis. Therefore, our results suggested that cuproptosis may be one of the underlying mechanisms of atherosclerosis. Meanwhile, we found that when cuproptosis occurred in HCAECs, the mRNA expression levels of intracellular ATP7B, MTF1, NLRP3, AOC3 and MT1M were up-regulated, which were consistent with the trends in the levels of these genes in the peripheral blood of atherosclerosis patients according to the bioinformatics analysis results, suggesting that the development of atherosclerosis may be accompanied by the continuous exacerbation of cuproptosis process. Finally, we further examined their role in cuproptosis by altering the expression levels of these genes. Our results manifested that knockdown of ATP7B and MTF1 genes in HCAECs intensified the process of cuproptosis (the expression levels of three signature proteins of cuproptosis were changed), while knockdown of NLRP3, AOC3 and MT1M genes in HCAECs did not significantly change. We hypothesized that this may be because ATP7B is the most important molecule regulating intracellular copper levels, and MTF1 could regulate copper levels by affecting the expression of ATP7B based on previous research reports [42]. Although the levels of NLRP3, AOC3 and MT1M were somewhat associated with intracellular copper levels, they may not directly regulate the levels of copper. However, the exact mechanism needs to be further revealed by future investigation. In addition, we also found 3 key genes for the molecular subtypes identification, which were related to the metabolic process or biological function of copper. Ceruloplasmin (CP) is a kind of copper oxidase, which could bind 40%–70 % copper in plasma and is an important regulatory molecule in copper transport process [52]. C–C motif chemokine ligand 8 (CCL8) is a member of the CC chemokine family. This family has been reported to regulate the progression of copper-induced atherosclerosis [30]. Dopamine-β-hydroxylase (DBH) is an enzyme that depends on copper, and the intracellular copper content could affect the expression level of DBH [53]. However, since these 3 genes were only differentially expressed in the 2 molecular subtype clusters, rather than between atherosclerosis group and control group, it is impossible to establish appropriate experimental models to verify their expression levels and functions.

Our study is the first to report the potential association between cuproptosis and atherosclerosis, and the total sample size included in our study is one of the largest in the field of atherosclerosis research so far. Therefore, this study brought convincing evidence for the exploration of new biomarkers for atherosclerosis diagnosis and molecular subtypes identification. Meanwhile, this study also opened up a novel direction for future investigation on the pathogenesis and targeted therapeutic drugs. However, we have to admit that this study has some limitations. First, the process of cuproptosis in cells contains multiple characteristic pathological changes. In this study, only the levels of the 3 most typical marker proteins were used to reflect the severity of cuproptosis, which may affect the rigor of the experimental results. Second, this study was only verified by cell experiments in vitro, lacking experimental verification of human arterial tissue or peripheral blood samples, and animal arterial tissue or peripheral blood samples. Third, the baseline characteristics of participants in the GEO datasets included in this study were not comprehensive enough. The GSE20129 dataset lacked baseline data of body mass index, age, comorbidity and gender. All datasets lacked information on medication and treatment, resulting in unclear baseline characteristics differences between atherosclerosis and control group. In addition, there was a lack of follow-up information for the participants, and hence it was not possible to analyze whether the differential CRGs identified in this study were correlated with the progression and prognosis of atherosclerosis patients. We hope the limitations can be solved in subsequent investigation.

Overall, this study found that endothelial cuproptosis may be a potential pathogenesis of atherosclerosis and CRGs might be promising markers for the molecular subtypes identification and diagnosis of atherosclerosis via bioinformatics analysis and experimental verification. Our research open up a novel investigation direction for atherosclerosis, and we look forward to future in-depth investigation based on this foundation to further reveal the pathogenesis of atherosclerosis and discover new diagnostic methods and treatment strategies.

## Author contribution statement

Mengxi Wang: Conceived and designed the experiments; Performed the experiments; Analyzed and interpreted the data; Wrote the paper.

Liying Cheng: Performed the experiments; Wrote the paper.

Xiaohu Chen: Conceived and designed the experiments; Wrote the paper.

Peng Yu: Conceived and designed the experiments; Wrote the paper>

Le Shen: Conceived and designed the experiments; Wrote the paper.

Qian Xiang: Performed the experiments; Analyzed and interpreted the data; Wrote the paper.

Yuhan Ding: Performed the experiments.

Ziwei Gao: Analyzed and interpreted the data.

Haitao Xie:Analyzed and interpreted the data.

## Data availability statement

Data included in article/supplementary material/referenced in article.

Supplementary content related to this article has been published online at [URL].

## Funding

This study was supported by the 10.13039/501100001809National Natural Science Foundation of China (82004308), the 10.13039/501100001809National Natural Science Foundation of China (81973824) and the National Administration of Traditional Chinese Medicine: 2019 Project of building evidence based practice capacity for TCM (2019XZZX-XXG004) and the Postgraduate Research & Practice Innovation Program of 10.13039/501100002949Jiangsu Province (KYCX22_1946).

## Declaration of competing interest

The authors declare that they have no known competing financial interests or personal relationships that could have appeared to influence the work reported in this paper.
