# Supplementary material for: Evaluation the role of cuproptosis-related genes in the pathogenesis, diagnosis and molecular subtypes identification of atherosclerosis
Source: Heliyon. 2023 Oct 18;9(10):e21158. doi: 10.1016/j.heliyon.2023.e21158 (PMC10622704; doi:10.1016/j.heliyon.2023.e21158)
Supplement: Multimedia component 1 [file mmc1.docx]

| **Supplementary Table S1.** **Baseline characteristics of** **participants in GSE20680 dataset.** | | | |
| --- | --- | --- | --- |
| **Variable** | **Control**  **(n=108)** | **Atherosclerosis**  **(n=87)** | ***P*-value** |
| Age (years) | 55.0 ± 11.0 | 63.0 ± 10.0 | < 0.001 |
| Gender (%) |  |  | 0.039 |
| Male | 55 (50.9%) | 58 (66.7%) |  |
| Female | 53 (49.1%) | 29 (33.3%) |  |
| Race (%) |  |  | 0.023 |
| Caucasian | 56 (51.9%) | 60 (69.0%) |  |
| Other race | 52 (48.1%) | 27 (31.0%) |  |
| BMI (kg/m^2^) | 32.0 ± 7.0 | 30.0 ± 6.0 | 0.098 |
| Current smoker (%) |  |  | 0.075 |
| Yes | 41 (38.0%) | 45 (51.7%) |  |
| No | 67 (62.0%) | 42 (48.3%) |  |
| Hypertension (%) |  |  | 0.084 |
| Yes | 67 (62.0%) | 65 (74.7%) |  |
| No | 41 (38.0%) | 22 (25.3%) |  |
| Dyslipidemia (%) |  |  | 0.039 |
| Yes | 55 (50.9%) | 58 (66.7%) |  |
| No | 53 (49.1%) | 29 (33.3%) |  |
| BMI, body mass index. | | | |

| **Supplementary Table S2.** **Baseline characteristics of participants in GSE20681 dataset.** | | | |
| --- | --- | --- | --- |
| **Variable** | **Control**  **(n=99)** | **Atherosclerosis**  **(n=99)** | ***P*-value** |
| Age (years) | 55.0 ± 12.0 | 62.0 ± 11.0 | < 0.001 |
| Gender (%) |  |  | > 0.999 |
| Male | 75 (75.8%) | 75 (75.8%) |  |
| Female | 24 (24.2%) | 24 (24.2%) |  |
| Race (%) |  |  | 0.166 |
| Caucasian | 85 (85.9%) | 92 (92.9%) |  |
| Other race | 14 (14.1%) | 7 (7.1%) |  |
| BMI (kg/m^2^) | 30.0 ± 7.0 | 30.0 ± 6.0 | 0.722 |
| Current smoker (%) |  |  | 0.074 |
| Yes | 14 (14.1%) | 25 (25.3%) |  |
| No | 85 (85.9%) | 74 (74.7%) |  |
| Hypertension (%) |  |  | 0.191 |
| Yes | 55 (55.6%) | 65 (65.7%) |  |
| No | 44 (44.4%) | 34 (34.3%) |  |
| Dyslipidemia (%) |  |  | 0.009 |
| Yes | 50 (50.5%) | 69 (69.7%) |  |
| No | 49 (49.5%) | 30 (30.3%) |  |
| BMI, body mass index. | | | |

| **Supplementary Table S3.** **Baseline characteristics of participants in GSE20129 dataset.** | | | |
| --- | --- | --- | --- |
| **Variable** | **Control**  **(n=86)** | **Atherosclerosis**  **(n=49)** | ***P*-value** |
| Race (%) |  |  | 0.648 |
| African American | 17 (19.8%) | 13 (26.5%) |  |
| Hispanic | 10 (11.6%) | 6 (12.2%) |  |
| Non-Hispanic white | 56 (65.1%) | 27 (55.1%) |  |
| Chinese | 3 (3.5%) | 3 (6.1%) |  |

| **Supplementary Table S4. Summary of CRGs symbol.** | | |
| --- | --- | --- |
| **CRGs symbol** | | |
| AOC3 | LOXL2 | SCO2 |
| ATOX1 | MAP2K1 | SLC25A3 |
| ATP7A | MAP2K2 | SLC31A1 |
| ATP7B | MT1A | SLC31A2 |
| CCL8 | MT1B | SOD1 |
| CCS | MT1E | TYR |
| CD274 | MT1F | UBE2D1 |
| CDKN2A | MT1G | UBE2D2 |
| COA6 | MT1H | UBE2D3 |
| COX11 | MT1M | UBE2D4 |
| COX17 | MT1X | ULK1 |
| CP | MT2A | ULK2 |
| DBH | MT-CO1 | VEGFA |
| DLAT | MT-CO2 | NFE2L2 |
| DLD | MTF1 | NLRP3 |
| FDX1 | PDE3B | LIPT2 |
| GLS | PDHA1 | DLST |
| HIST1H3A | PDHB | DBT |
| LIAS | PDK1 | GCSH |
| LIPT1 | SCO1 |  |

| **Supplementary Table S5. Summary of primer sequences.** | | | |
| --- | --- | --- | --- |
| **Name** | **Species** | **Forward primer** | **Reverse primer** |
| AOC3 | Human | CCAAGGATCTTTGACGTTCGC | CTCCATCCACATAGCGGGTC |
| MT1M | Human | AGCAGTCGCTCCATTTATC | CAGTTCTCCAACGTCCCT |
| MTF1 | Human | CACAGTCCAGACAACAACATCA | GCACCAGTCCGTTTTTATCCAC |
| NLRP3 | Human | CGTGAGTCCCATTAAGATGGAGT | CCCGACAGTGGATATAGAACAGA |
| ATP7B | Human | GCCAGCATTGCAGAAGGAAAG | TGATAAGTGATGACGGCCTCT |
| IL-1β | Human | ATGATGGCTTATTACAGTGGCAA | GTCGGAGATTCGTAGCTGGA |
| IL-6 | Human | ACTCACCTCTTCAGAACGAATTG | CCATCTTTGGAAGGTTCAGGTTG |
| TNF-α | Human | GAGGCCAAGCCCTGGTATG | CGGGCCGATTGATCTCAGC |
| GAPDH | Human | GGACCTGACCTGCCGTCTAG | GTAGCCCAGGATGCCCTTGA |

| **Supplementary Table S6. Details of 5 A-CRGs.** | | |
| --- | --- | --- |
| **Gene symbol** | **Encoded protein** | **Roles in cuproptosis** |
| ATP7B | ATPase copper transporting beta | Regulation of the copper conte1nt in cells |
| MTF1 | Metal regulatory transcription factor 1 | Transfer from cytoplasm to nucleus to maintain copper homeostasis in cells |
| NLRP3 | NOD-like receptor thermal protein domain associated protein 3 | Expression level was positively correlated with copper content and ATP7A expression level |
| AOC3 | Amine oxidase copper containing 3 | Expression level was positively correlated with copper content |
| MT1M | Metallothionein 1M | Bind copper and regulation of copper homeostasis in cells |

| **Supplementary Table S7. Details of 3 M-CRGs.** | | |
| --- | --- | --- |
| **Gene symbol** | **Encoded protein** | **Roles in cuproptosis** |
| CP | Ceruloplasmin | Bind copper in plasma and  regulation of copper transport process |
| CCL8 | C-C motif chemokine ligand 8 | Expression level was correlated with copper content |
| DBH | Dopamine-β-hydroxylase | A copper-dependent enzyme and copper content could affect its expression level |

| **Supplementary Table S8. Interactive relationship of each mRNA, miRNA and lncRNA.** | | |
| --- | --- | --- |
| **Name of miRNA** | **Name of interacting**  **mRNA or lncRNA** | **Interaction type** |
| hsa-miR-345-5p | CCL8 | miRNA-mRNA |
| hsa-miR-1268a | DBH | miRNA-mRNA |
| hsa-miR-922 | MTF1 | miRNA-mRNA |
| hsa-miR-28-5p | MTF1 | miRNA-mRNA |
| hsa-miR-524-5p | CP | miRNA-mRNA |
| hsa-miR-302e | MTF1 | miRNA-mRNA |
| hsa-miR-195-3p | MTF1 | miRNA-mRNA |
| hsa-miR-23b-3p | MTF1 | miRNA-mRNA |
| hsa-miR-4282 | NLRP3 | miRNA-mRNA |
| hsa-miR-708-5p | MTF1 | miRNA-mRNA |
| hsa-miR-1238-3p | ATP7B | miRNA-mRNA |
| hsa-miR-660-5p | AOC3 | miRNA-mRNA |
| hsa-miR-569 | CP | miRNA-mRNA |
| hsa-miR-125a-5p | MTF1 | miRNA-mRNA |
| hsa-miR-548w | MTF1 | miRNA-mRNA |
| hsa-miR-148a-3p | MTF1 | miRNA-mRNA |
| hsa-miR-1267 | CP | miRNA-mRNA |
| hsa-miR-541-3p | DBH | miRNA-mRNA |
| hsa-miR-450b-5p | CP | miRNA-mRNA |
| hsa-miR-148b-5p | NLRP3 | miRNA-mRNA |
| hsa-miR-367-3p | MTF1 | miRNA-mRNA |
| hsa-miR-221-5p | AOC3 | miRNA-mRNA |
| hsa-miR-24-3p | MTF1 | miRNA-mRNA |
| hsa-miR-577 | MTF1 | miRNA-mRNA |
| hsa-miR-376a-5p | ATP7B | miRNA-mRNA |
| hsa-miR-338-5p | MTF1 | miRNA-mRNA |
| hsa-miR-106b-5p | MTF1 | miRNA-mRNA |
| hsa-let-7f-1-3p | MTF1 | miRNA-mRNA |
| hsa-miR-3065-5p | CCL8 | miRNA-mRNA |
| hsa-miR-185-5p | ATP7B | miRNA-mRNA |
| hsa-miR-802 | MTF1 | miRNA-mRNA |
| hsa-miR-3133 | CP | miRNA-mRNA |
| hsa-miR-485-5p | MTF1 | miRNA-mRNA |
| hsa-miR-149-3p | MTF1 | miRNA-mRNA |
| hsa-miR-22-5p | ATP7B | miRNA-mRNA |
| hsa-miR-518c-5p | MTF1 | miRNA-mRNA |
| hsa-miR-214-5p | ATP7B | miRNA-mRNA |
| hsa-miR-373-5p | MTF1 | miRNA-mRNA |
| hsa-miR-186-5p | MTF1 | miRNA-mRNA |
| hsa-miR-302a-3p | MTF1 | miRNA-mRNA |
| hsa-miR-520d-3p | MTF1 | miRNA-mRNA |
| hsa-miR-1207-5p | ATP7B | miRNA-mRNA |
| hsa-miR-15b-3p | CP | miRNA-mRNA |
| hsa-miR-944 | CCL8 | miRNA-mRNA |
| hsa-miR-1255b-5p | AOC3 | miRNA-mRNA |
| hsa-miR-3197 | NLRP3 | miRNA-mRNA |
| hsa-miR-4255 | CP | miRNA-mRNA |
| hsa-miR-548d-5p | MTF1 | miRNA-mRNA |
| hsa-miR-302d-3p | MTF1 | miRNA-mRNA |
| hsa-miR-543 | CP | miRNA-mRNA |
| hsa-miR-149-3p | AOC3 | miRNA-mRNA |
| hsa-miR-520a-3p | MTF1 | miRNA-mRNA |
| hsa-miR-610 | NLRP3 | miRNA-mRNA |
| hsa-miR-578 | CP | miRNA-mRNA |
| hsa-miR-545-3p | MTF1 | miRNA-mRNA |
| hsa-miR-2110 | CCL8 | miRNA-mRNA |
| hsa-miR-421 | ATP7B | miRNA-mRNA |
| hsa-miR-3163 | ATP7B | miRNA-mRNA |
| hsa-miR-4327 | MTF1 | miRNA-mRNA |
| hsa-miR-3140-3p | MTF1 | miRNA-mRNA |
| hsa-miR-302c-5p | MT1M | miRNA-mRNA |
| hsa-miR-548a-5p | MTF1 | miRNA-mRNA |
| hsa-miR-762 | AOC3 | miRNA-mRNA |
| hsa-miR-4310 | MTF1 | miRNA-mRNA |
| hsa-miR-30b-3p | MTF1 | miRNA-mRNA |
| hsa-miR-520c-3p | MTF1 | miRNA-mRNA |
| hsa-miR-24-3p | MT1M | miRNA-mRNA |
| hsa-miR-548l | MTF1 | miRNA-mRNA |
| hsa-miR-1-3p | AOC3 | miRNA-mRNA |
| hsa-miR-3157-5p | CP | miRNA-mRNA |
| hsa-miR-548c-5p | MTF1 | miRNA-mRNA |
| hsa-miR-4267 | AOC3 | miRNA-mRNA |
| hsa-miR-4251 | MTF1 | miRNA-mRNA |
| hsa-miR-556-3p | CP | miRNA-mRNA |
| hsa-miR-4291 | MTF1 | miRNA-mRNA |
| hsa-miR-4303 | MTF1 | miRNA-mRNA |
| hsa-miR-365a-3p | MTF1 | miRNA-mRNA |
| hsa-miR-559 | MTF1 | miRNA-mRNA |
| hsa-miR-374b-5p | CCL8 | miRNA-mRNA |
| hsa-miR-150-5p | AOC3 | miRNA-mRNA |
| hsa-miR-18a-3p | MTF1 | miRNA-mRNA |
| hsa-miR-421 | MTF1 | miRNA-mRNA |
| hsa-miR-888-5p | CP | miRNA-mRNA |
| hsa-miR-661 | MTF1 | miRNA-mRNA |
| hsa-miR-32-5p | MTF1 | miRNA-mRNA |
| hsa-miR-548p | MTF1 | miRNA-mRNA |
| hsa-miR-4269 | MTF1 | miRNA-mRNA |
| hsa-miR-204-5p | DBH | miRNA-mRNA |
| hsa-miR-4307 | CP | miRNA-mRNA |
| hsa-miR-181c-5p | CCL8 | miRNA-mRNA |
| hsa-miR-10b-5p | MTF1 | miRNA-mRNA |
| hsa-miR-599 | CP | miRNA-mRNA |
| hsa-miR-1265 | CCL8 | miRNA-mRNA |
| hsa-miR-548a-3p | MTF1 | miRNA-mRNA |
| hsa-miR-3148 | MTF1 | miRNA-mRNA |
| hsa-miR-21-3p | MTF1 | miRNA-mRNA |
| hsa-miR-214-3p | MTF1 | miRNA-mRNA |
| hsa-miR-4293 | MTF1 | miRNA-mRNA |
| hsa-miR-146a-3p | CP | miRNA-mRNA |
| hsa-miR-448 | MTF1 | miRNA-mRNA |
| hsa-miR-3125 | ATP7B | miRNA-mRNA |
| hsa-miR-4256 | MTF1 | miRNA-mRNA |
| hsa-miR-1255a | AOC3 | miRNA-mRNA |
| hsa-miR-4271 | MTF1 | miRNA-mRNA |
| hsa-miR-765 | DBH | miRNA-mRNA |
| hsa-miR-3176 | MTF1 | miRNA-mRNA |
| hsa-miR-22-3p | MTF1 | miRNA-mRNA |
| hsa-miR-557 | MTF1 | miRNA-mRNA |
| hsa-miR-1911-3p | AOC3 | miRNA-mRNA |
| hsa-miR-1305 | NLRP3 | miRNA-mRNA |
| hsa-miR-1208 | CP | miRNA-mRNA |
| hsa-miR-1297 | MT1M | miRNA-mRNA |
| hsa-miR-548x-3p | ATP7B | miRNA-mRNA |
| hsa-miR-374b-3p | MTF1 | miRNA-mRNA |
| hsa-miR-1278 | MTF1 | miRNA-mRNA |
| hsa-miR-92a-1-5p | ATP7B | miRNA-mRNA |
| hsa-miR-1237-3p | MTF1 | miRNA-mRNA |
| hsa-miR-181a-5p | CCL8 | miRNA-mRNA |
| hsa-miR-548t-5p | MTF1 | miRNA-mRNA |
| hsa-miR-544b | ATP7B | miRNA-mRNA |
| hsa-miR-506-3p | MTF1 | miRNA-mRNA |
| hsa-miR-519e-5p | MTF1 | miRNA-mRNA |
| hsa-miR-548i | NLRP3 | miRNA-mRNA |
| hsa-miR-649 | MTF1 | miRNA-mRNA |
| hsa-miR-582-3p | CP | miRNA-mRNA |
| hsa-miR-940 | AOC3 | miRNA-mRNA |
| hsa-miR-302b-3p | MTF1 | miRNA-mRNA |
| hsa-miR-20b-5p | MTF1 | miRNA-mRNA |
| hsa-miR-1248 | MTF1 | miRNA-mRNA |
| hsa-miR-556-5p | NLRP3 | miRNA-mRNA |
| hsa-miR-10a-5p | MTF1 | miRNA-mRNA |
| hsa-miR-4328 | CP | miRNA-mRNA |
| hsa-miR-3134 | MTF1 | miRNA-mRNA |
| hsa-miR-3190-3p | MTF1 | miRNA-mRNA |
| hsa-miR-129-5p | MTF1 | miRNA-mRNA |
| hsa-let-7a-3p | MTF1 | miRNA-mRNA |
| hsa-miR-505-5p | MTF1 | miRNA-mRNA |
| hsa-miR-223-3p | NLRP3 | miRNA-mRNA |
| hsa-miR-2115-3p | MTF1 | miRNA-mRNA |
| hsa-miR-587 | CP | miRNA-mRNA |
| hsa-miR-592 | CP | miRNA-mRNA |
| hsa-miR-548h-5p | MTF1 | miRNA-mRNA |
| hsa-miR-449c-3p | MTF1 | miRNA-mRNA |
| hsa-miR-30b-3p | ATP7B | miRNA-mRNA |
| hsa-miR-1305 | CCL8 | miRNA-mRNA |
| hsa-miR-4261 | CP | miRNA-mRNA |
| hsa-miR-106a-5p | MTF1 | miRNA-mRNA |
| hsa-miR-607 | MTF1 | miRNA-mRNA |
| hsa-miR-647 | MTF1 | miRNA-mRNA |
| hsa-miR-33a-3p | CP | miRNA-mRNA |
| hsa-miR-548b-5p | NLRP3 | miRNA-mRNA |
| hsa-miR-125b-1-3p | MTF1 | miRNA-mRNA |
| hsa-miR-761 | MTF1 | miRNA-mRNA |
| hsa-miR-588 | AOC3 | miRNA-mRNA |
| hsa-miR-3202 | MTF1 | miRNA-mRNA |
| hsa-miR-211-5p | DBH | miRNA-mRNA |
| hsa-let-7b-3p | MTF1 | miRNA-mRNA |
| hsa-miR-148b-3p | MTF1 | miRNA-mRNA |
| hsa-miR-633 | CP | miRNA-mRNA |
| hsa-miR-488-3p | MTF1 | miRNA-mRNA |
| hsa-miR-10b-3p | MTF1 | miRNA-mRNA |
| hsa-miR-93-5p | MTF1 | miRNA-mRNA |
| hsa-miR-3190-5p | MTF1 | miRNA-mRNA |
| hsa-miR-125b-5p | MTF1 | miRNA-mRNA |
| hsa-miR-363-3p | MTF1 | miRNA-mRNA |
| hsa-miR-617 | AOC3 | miRNA-mRNA |
| hsa-miR-361-3p | CP | miRNA-mRNA |
| hsa-miR-22-3p | NLRP3 | miRNA-mRNA |
| hsa-miR-559 | NLRP3 | miRNA-mRNA |
| hsa-miR-548w | NLRP3 | miRNA-mRNA |
| hsa-miR-939-5p | MTF1 | miRNA-mRNA |
| hsa-miR-654-5p | DBH | miRNA-mRNA |
| hsa-miR-877-5p | ATP7B | miRNA-mRNA |
| hsa-miR-449c-5p | MTF1 | miRNA-mRNA |
| hsa-miR-507 | MTF1 | miRNA-mRNA |
| hsa-miR-1290 | NLRP3 | miRNA-mRNA |
| hsa-miR-765 | AOC3 | miRNA-mRNA |
| hsa-miR-181b-5p | CCL8 | miRNA-mRNA |
| hsa-miR-30c-2-3p | ATP7B | miRNA-mRNA |
| hsa-miR-491-3p | MTF1 | miRNA-mRNA |
| hsa-miR-3125 | MTF1 | miRNA-mRNA |
| hsa-miR-570-3p | ATP7B | miRNA-mRNA |
| hsa-miR-3194-5p | DBH | miRNA-mRNA |
| hsa-miR-876-5p | MTF1 | miRNA-mRNA |
| hsa-miR-3133 | MTF1 | miRNA-mRNA |
| hsa-miR-548c-5p | NLRP3 | miRNA-mRNA |
| hsa-miR-92b-3p | MTF1 | miRNA-mRNA |
| hsa-miR-539-5p | MTF1 | miRNA-mRNA |
| hsa-miR-3180-5p | CP | miRNA-mRNA |
| hsa-miR-133b | ATP7B | miRNA-mRNA |
| hsa-miR-548h-5p | NLRP3 | miRNA-mRNA |
| hsa-miR-186-5p | CP | miRNA-mRNA |
| hsa-miR-548d-5p | NLRP3 | miRNA-mRNA |
| hsa-miR-548k | MTF1 | miRNA-mRNA |
| hsa-miR-4325 | MTF1 | miRNA-mRNA |
| hsa-miR-105-5p | CP | miRNA-mRNA |
| hsa-miR-548a-5p | NLRP3 | miRNA-mRNA |
| hsa-miR-25-3p | MTF1 | miRNA-mRNA |
| hsa-miR-1207-5p | MTF1 | miRNA-mRNA |
| hsa-miR-875-3p | MTF1 | miRNA-mRNA |
| hsa-miR-3138 | ATP7B | miRNA-mRNA |
| hsa-miR-770-5p | MTF1 | miRNA-mRNA |
| hsa-miR-92a-2-5p | CP | miRNA-mRNA |
| hsa-miR-484 | ATP7B | miRNA-mRNA |
| hsa-miR-576-5p | ATP7B | miRNA-mRNA |
| hsa-miR-548i | MTF1 | miRNA-mRNA |
| hsa-miR-144-5p | CP | miRNA-mRNA |
| hsa-miR-548t-5p | ATP7B | miRNA-mRNA |
| hsa-miR-515-5p | MTF1 | miRNA-mRNA |
| hsa-miR-570-3p | CP | miRNA-mRNA |
| hsa-miR-548c-3p | ATP7B | miRNA-mRNA |
| hsa-miR-548b-5p | MTF1 | miRNA-mRNA |
| hsa-miR-625-5p | DBH | miRNA-mRNA |
| hsa-miR-373-3p | MTF1 | miRNA-mRNA |
| hsa-miR-1290 | ATP7B | miRNA-mRNA |
| hsa-miR-187-5p | NLRP3 | miRNA-mRNA |
| hsa-miR-4264 | CCL8 | miRNA-mRNA |
| hsa-miR-1303 | AOC3 | miRNA-mRNA |
| hsa-miR-2115-3p | AOC3 | miRNA-mRNA |
| hsa-miR-513a-5p | MTF1 | miRNA-mRNA |
| hsa-miR-149-5p | MTF1 | miRNA-mRNA |
| hsa-miR-145-5p | MTF1 | miRNA-mRNA |
| hsa-miR-130a-5p | CP | miRNA-mRNA |
| hsa-miR-16-2-3p | MTF1 | miRNA-mRNA |
| hsa-miR-23a-3p | MTF1 | miRNA-mRNA |
| hsa-miR-1909-3p | DBH | miRNA-mRNA |
| hsa-miR-133b | LINC00487 | lncRNA-miRNA |
| hsa-miR-133b | C14orf182 | lncRNA-miRNA |
| hsa-miR-133b | SNHG14 | lncRNA-miRNA |
| hsa-miR-133b | BACE2-IT1 | lncRNA-miRNA |
| hsa-miR-133b | OSBPL10-AS1 | lncRNA-miRNA |
| hsa-miR-133b | LINC00271 | lncRNA-miRNA |
| hsa-miR-133b | KCNQ1OT1 | lncRNA-miRNA |
| hsa-miR-761 | C21orf90 | lncRNA-miRNA |
| hsa-miR-761 | LINC00176 | lncRNA-miRNA |
| hsa-miR-761 | C1orf132 | lncRNA-miRNA |
| hsa-miR-761 | ZNRD1-AS1 | lncRNA-miRNA |
| hsa-miR-761 | C14orf182 | lncRNA-miRNA |
| hsa-miR-761 | TTTY4 | lncRNA-miRNA |
| hsa-miR-761 | TTTY4C | lncRNA-miRNA |
| hsa-miR-761 | XIST | lncRNA-miRNA |
| hsa-miR-761 | ATXN8OS | lncRNA-miRNA |
| hsa-miR-761 | TTTY4B | lncRNA-miRNA |
| hsa-miR-761 | FAM138A | lncRNA-miRNA |
| hsa-miR-761 | FAM138E | lncRNA-miRNA |
| hsa-miR-761 | SHANK3 | lncRNA-miRNA |
| hsa-miR-761 | KCNQ1OT1 | lncRNA-miRNA |
| hsa-miR-761 | MIR4313 | lncRNA-miRNA |
| hsa-miR-23b-3p | ZNRD1-AS1 | lncRNA-miRNA |
| hsa-miR-23b-3p | MUC19 | lncRNA-miRNA |
| hsa-miR-23b-3p | SNHG14 | lncRNA-miRNA |
| hsa-miR-23b-3p | TTTY4 | lncRNA-miRNA |
| hsa-miR-23b-3p | TTTY4C | lncRNA-miRNA |
| hsa-miR-23b-3p | XIST | lncRNA-miRNA |
| hsa-miR-23b-3p | ZFY-AS1 | lncRNA-miRNA |
| hsa-miR-23b-3p | TTTY4B | lncRNA-miRNA |
| hsa-miR-23b-3p | NEAT1 | lncRNA-miRNA |
| hsa-miR-23b-3p | KCNQ1OT1 | lncRNA-miRNA |
| hsa-miR-23b-3p | ITPK1-AS1 | lncRNA-miRNA |
| hsa-miR-1297 | PCBP1-AS1 | lncRNA-miRNA |
| hsa-miR-1297 | LINC00238 | lncRNA-miRNA |
| hsa-miR-1297 | VWC2L-IT1 | lncRNA-miRNA |
| hsa-miR-1297 | ARPP21-AS1 | lncRNA-miRNA |
| hsa-miR-1297 | WASIR2 | lncRNA-miRNA |
| hsa-miR-1297 | GAS5 | lncRNA-miRNA |
| hsa-miR-1297 | ADAMTS9-AS1 | lncRNA-miRNA |
| hsa-miR-1297 | TUG1 | lncRNA-miRNA |
| hsa-miR-1297 | KCNQ1OT1 | lncRNA-miRNA |
| hsa-miR-129-5p | TPTEP1 | lncRNA-miRNA |
| hsa-miR-129-5p | KCNA3 | lncRNA-miRNA |
| hsa-miR-129-5p | TTTY8B | lncRNA-miRNA |
| hsa-miR-129-5p | SNHG12 | lncRNA-miRNA |
| hsa-miR-129-5p | MUC19 | lncRNA-miRNA |
| hsa-miR-129-5p | UCA1 | lncRNA-miRNA |
| hsa-miR-129-5p | MEG3 | lncRNA-miRNA |
| hsa-miR-129-5p | HLA-F-AS1 | lncRNA-miRNA |
| hsa-miR-129-5p | SNHG14 | lncRNA-miRNA |
| hsa-miR-129-5p | TTTY16 | lncRNA-miRNA |
| hsa-miR-129-5p | HOTAIR | lncRNA-miRNA |
| hsa-miR-129-5p | LINC00276 | lncRNA-miRNA |
| hsa-miR-129-5p | HCG18 | lncRNA-miRNA |
| hsa-miR-129-5p | DLX6-AS1 | lncRNA-miRNA |
| hsa-miR-129-5p | ERVH48-1 | lncRNA-miRNA |
| hsa-miR-129-5p | C10orf40 | lncRNA-miRNA |
| hsa-miR-129-5p | NEAT1 | lncRNA-miRNA |
| hsa-miR-129-5p | MALAT1 | lncRNA-miRNA |
| hsa-miR-129-5p | PCAT1 | lncRNA-miRNA |
| hsa-miR-129-5p | KCNQ1OT1 | lncRNA-miRNA |
| hsa-miR-302e | HNRNPU-AS1 | lncRNA-miRNA |
| hsa-miR-302e | COX10-AS1 | lncRNA-miRNA |
| hsa-miR-302e | SPAG5-AS1 | lncRNA-miRNA |
| hsa-miR-302e | TTTY15 | lncRNA-miRNA |
| hsa-miR-302e | SEC62-AS1 | lncRNA-miRNA |
| hsa-miR-302e | LINC00293 | lncRNA-miRNA |
| hsa-miR-302e | GRM5-AS1 | lncRNA-miRNA |
| hsa-miR-302e | KCNQ1OT1 | lncRNA-miRNA |
| hsa-miR-520a-3p | COX10-AS1 | lncRNA-miRNA |
| hsa-miR-520a-3p | SPAG5-AS1 | lncRNA-miRNA |
| hsa-miR-520a-3p | XIST | lncRNA-miRNA |
| hsa-miR-520a-3p | TTTY15 | lncRNA-miRNA |
| hsa-miR-520a-3p | PVRL3-AS1 | lncRNA-miRNA |
| hsa-miR-520a-3p | LINC00293 | lncRNA-miRNA |
| hsa-miR-520a-3p | KCNQ1OT1 | lncRNA-miRNA |
| hsa-miR-520c-3p | C12orf36 | lncRNA-miRNA |
| hsa-miR-520c-3p | COX10-AS1 | lncRNA-miRNA |
| hsa-miR-520c-3p | SPAG5-AS1 | lncRNA-miRNA |
| hsa-miR-520c-3p | XIST | lncRNA-miRNA |
| hsa-miR-520c-3p | TTTY15 | lncRNA-miRNA |
| hsa-miR-520c-3p | PVRL3-AS1 | lncRNA-miRNA |
| hsa-miR-520c-3p | LINC00293 | lncRNA-miRNA |
| hsa-miR-520c-3p | KCNQ1OT1 | lncRNA-miRNA |
| hsa-miR-520d-3p | C12orf36 | lncRNA-miRNA |
| hsa-miR-520d-3p | COX10-AS1 | lncRNA-miRNA |
| hsa-miR-520d-3p | SPAG5-AS1 | lncRNA-miRNA |
| hsa-miR-520d-3p | XIST | lncRNA-miRNA |
| hsa-miR-520d-3p | TTTY15 | lncRNA-miRNA |
| hsa-miR-520d-3p | PVRL3-AS1 | lncRNA-miRNA |
| hsa-miR-520d-3p | LINC00293 | lncRNA-miRNA |
| hsa-miR-520d-3p | KCNQ1OT1 | lncRNA-miRNA |
| hsa-miR-148a-3p | LINC00221 | lncRNA-miRNA |
| hsa-miR-148a-3p | SNHG14 | lncRNA-miRNA |
| hsa-miR-148a-3p | DNM3OS | lncRNA-miRNA |
| hsa-miR-148a-3p | ITCH-IT1 | lncRNA-miRNA |
| hsa-miR-148a-3p | HOTAIRM1 | lncRNA-miRNA |
| hsa-miR-148a-3p | OIP5-AS1 | lncRNA-miRNA |
| hsa-miR-148a-3p | KCNQ1OT1 | lncRNA-miRNA |
| hsa-miR-148a-3p | MIR4313 | lncRNA-miRNA |
| hsa-miR-148b-3p | C21orf88 | lncRNA-miRNA |
| hsa-miR-148b-3p | LINC00221 | lncRNA-miRNA |
| hsa-miR-148b-3p | ZNRD1-AS1 | lncRNA-miRNA |
| hsa-miR-148b-3p | SNHG14 | lncRNA-miRNA |
| hsa-miR-148b-3p | TTTY10 | lncRNA-miRNA |
| hsa-miR-148b-3p | DNM3OS | lncRNA-miRNA |
| hsa-miR-148b-3p | ITCH-IT1 | lncRNA-miRNA |
| hsa-miR-148b-3p | MCCC1-AS1 | lncRNA-miRNA |
| hsa-miR-148b-3p | OIP5-AS1 | lncRNA-miRNA |
| hsa-miR-148b-3p | KCNQ1OT1 | lncRNA-miRNA |
| hsa-miR-148b-3p | MIR4313 | lncRNA-miRNA |
| hsa-miR-20b-5p | AGAP11 | lncRNA-miRNA |
| hsa-miR-20b-5p | C12orf36 | lncRNA-miRNA |
| hsa-miR-20b-5p | COX10-AS1 | lncRNA-miRNA |
| hsa-miR-20b-5p | HCG11 | lncRNA-miRNA |
| hsa-miR-20b-5p | TTTY10 | lncRNA-miRNA |
| hsa-miR-20b-5p | HOTAIRM1 | lncRNA-miRNA |
| hsa-miR-20b-5p | TTTY15 | lncRNA-miRNA |
| hsa-miR-20b-5p | BCYRN1 | lncRNA-miRNA |
| hsa-miR-20b-5p | HNF1A-AS1 | lncRNA-miRNA |
| hsa-miR-20b-5p | PVRL3-AS1 | lncRNA-miRNA |
| hsa-miR-20b-5p | VCAN-AS1 | lncRNA-miRNA |
| hsa-miR-20b-5p | LINC00293 | lncRNA-miRNA |
| hsa-miR-20b-5p | KCNQ1OT1 | lncRNA-miRNA |
| hsa-miR-24-3p | TPTEP1 | lncRNA-miRNA |
| hsa-miR-24-3p | C21orf90 | lncRNA-miRNA |
| hsa-miR-24-3p | C21orf67 | lncRNA-miRNA |
| hsa-miR-24-3p | LINC00518 | lncRNA-miRNA |
| hsa-miR-24-3p | C14orf182 | lncRNA-miRNA |
| hsa-miR-24-3p | HCG20 | lncRNA-miRNA |
| hsa-miR-24-3p | C1orf143 | lncRNA-miRNA |
| hsa-miR-24-3p | BCYRN1 | lncRNA-miRNA |
| hsa-miR-24-3p | DNAJC3-AS1 | lncRNA-miRNA |
| hsa-miR-24-3p | RMST | lncRNA-miRNA |
| hsa-miR-24-3p | KCNQ1OT1 | lncRNA-miRNA |
| hsa-miR-24-3p | DIO3OS | lncRNA-miRNA |
| hsa-miR-507 | MUC19 | lncRNA-miRNA |
| hsa-miR-507 | XIST | lncRNA-miRNA |
| hsa-miR-507 | LINC00461 | lncRNA-miRNA |
| hsa-miR-507 | NEAT1 | lncRNA-miRNA |
| hsa-miR-507 | MALAT1 | lncRNA-miRNA |
| hsa-miR-507 | LINC00293 | lncRNA-miRNA |
| hsa-miR-507 | KCNQ1OT1 | lncRNA-miRNA |
| hsa-miR-22-3p | FAM201A | lncRNA-miRNA |
| hsa-miR-22-3p | OIP5-AS1 | lncRNA-miRNA |
| hsa-miR-22-3p | SHANK3 | lncRNA-miRNA |
| hsa-miR-22-3p | DYX1C1-CCPG1 | lncRNA-miRNA |
| hsa-miR-22-3p | KCNQ1OT1 | lncRNA-miRNA |
| hsa-miR-449c-5p | C21orf90 | lncRNA-miRNA |
| hsa-miR-449c-5p | XIST | lncRNA-miRNA |
| hsa-miR-449c-5p | KCNQ1OT1 | lncRNA-miRNA |
| hsa-miR-449c-5p | ARHGAP5-AS1 | lncRNA-miRNA |
| hsa-miR-125a-5p | MUC19 | lncRNA-miRNA |
| hsa-miR-125a-5p | LINC00475 | lncRNA-miRNA |
| hsa-miR-125a-5p | TTTY10 | lncRNA-miRNA |
| hsa-miR-125a-5p | MIR497HG | lncRNA-miRNA |
| hsa-miR-125a-5p | POU6F2-AS2 | lncRNA-miRNA |
| hsa-miR-125a-5p | GNAS-AS1 | lncRNA-miRNA |
| hsa-miR-125a-5p | SHANK3 | lncRNA-miRNA |
| hsa-miR-125a-5p | KCNQ1OT1 | lncRNA-miRNA |
| hsa-miR-125b-5p | MUC19 | lncRNA-miRNA |
| hsa-miR-125b-5p | LINC00475 | lncRNA-miRNA |
| hsa-miR-125b-5p | TTTY10 | lncRNA-miRNA |
| hsa-miR-125b-5p | MIR497HG | lncRNA-miRNA |
| hsa-miR-125b-5p | POU6F2-AS2 | lncRNA-miRNA |
| hsa-miR-125b-5p | GNAS-AS1 | lncRNA-miRNA |
| hsa-miR-125b-5p | PRMT5-AS1 | lncRNA-miRNA |
| hsa-miR-125b-5p | SHANK3 | lncRNA-miRNA |
| hsa-miR-125b-5p | KCNQ1OT1 | lncRNA-miRNA |
| hsa-miR-10a-5p | STK24-AS1 | lncRNA-miRNA |
| hsa-miR-10a-5p | LINC00475 | lncRNA-miRNA |
| hsa-miR-10a-5p | FAM66C | lncRNA-miRNA |
| hsa-miR-10a-5p | HCG20 | lncRNA-miRNA |
| hsa-miR-10a-5p | TTTY15 | lncRNA-miRNA |
| hsa-miR-10a-5p | ARAP1-AS2 | lncRNA-miRNA |
| hsa-miR-10a-5p | SHANK3 | lncRNA-miRNA |
| hsa-miR-10a-5p | KCNQ1OT1 | lncRNA-miRNA |
| hsa-miR-10a-5p | VENTXP1 | lncRNA-miRNA |
| hsa-miR-363-3p | CXorf24 | lncRNA-miRNA |
| hsa-miR-363-3p | XIST | lncRNA-miRNA |
| hsa-miR-363-3p | LINC00293 | lncRNA-miRNA |
| hsa-miR-363-3p | KCNQ1OT1 | lncRNA-miRNA |
| hsa-miR-33a-3p | TUG1 | lncRNA-miRNA |
| hsa-miR-33a-3p | KCNQ1OT1 | lncRNA-miRNA |

**Supplementary Figures**
**
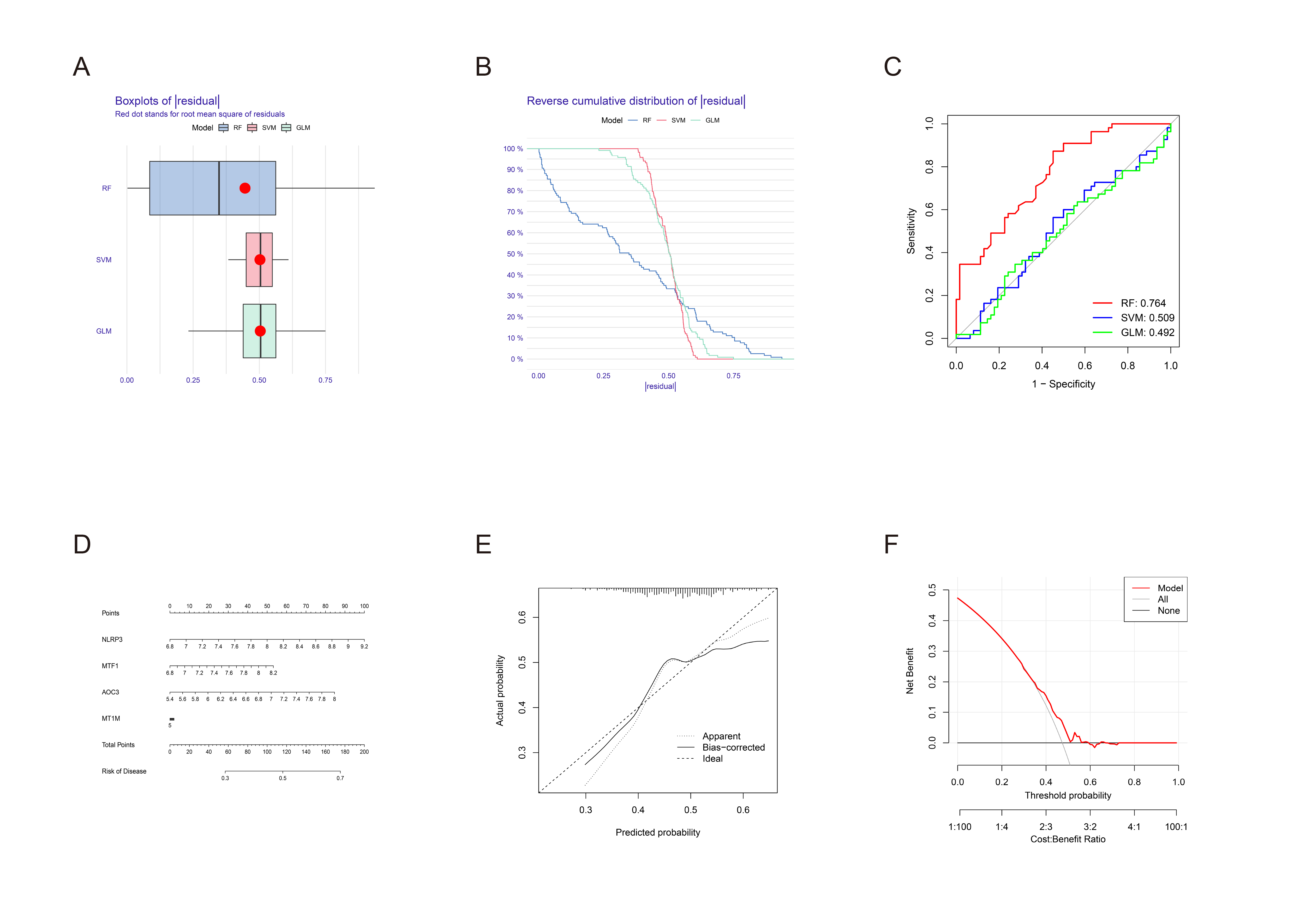
**

**Supplementary Fig.S1.** Assessment of the value of 4 A-CRGs in atherosclerosis diagnosis. (A-C) Residual boxplot, cumulative residual distribution curve and ROC curve of machine learning algorithm. (D) Nomogram of 4 A-CRGs. (E-F) Calibration curve and DCA of the nomogram.


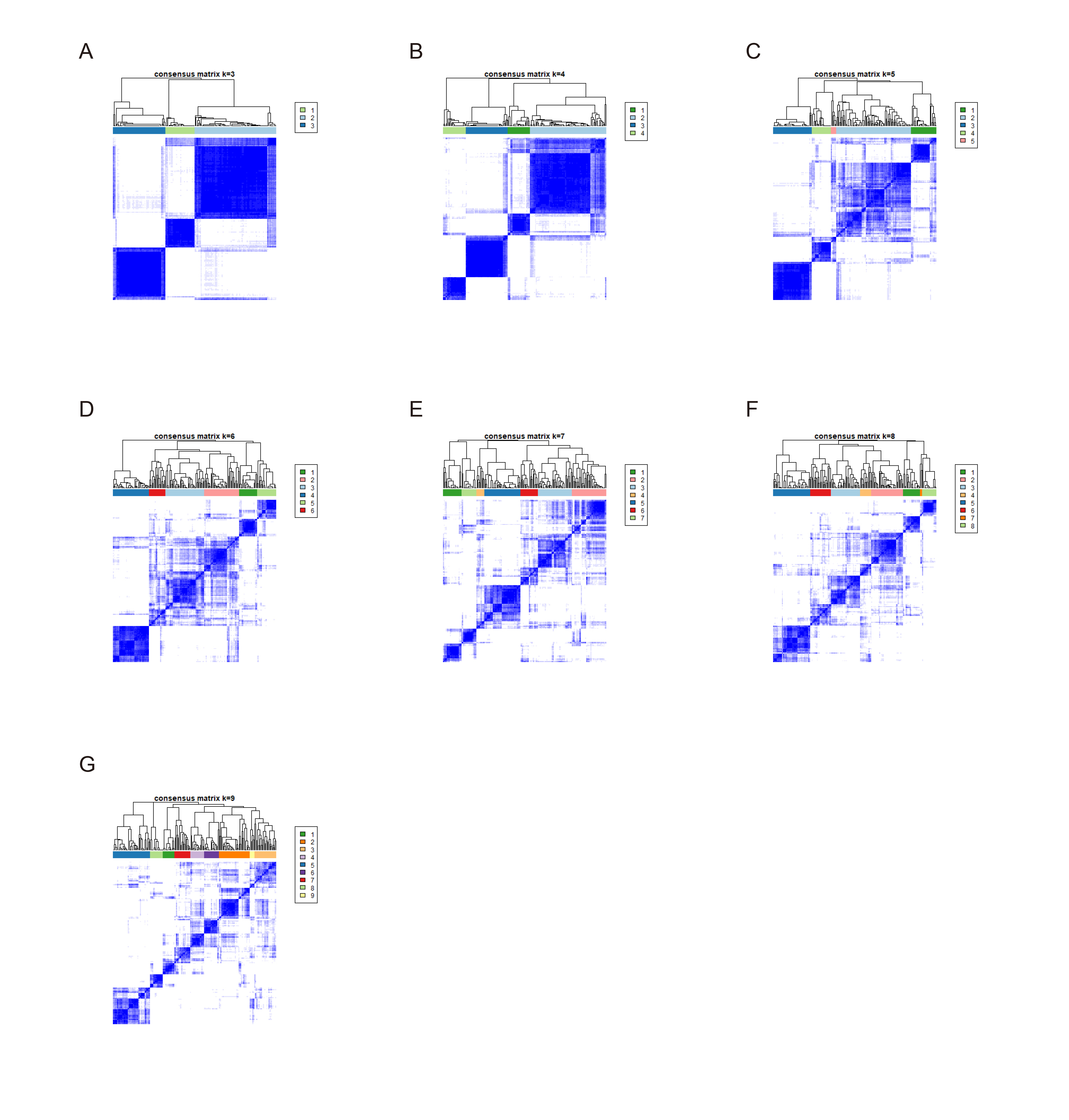


**Supplementary Fig.S2.** The consensus clustering matrix when k = 3-9. (A) k = 3. (B) k = 4. (C) k = 5. (D) k = 6. (E) k = 7. (F) k = 8. (G) k = 9.


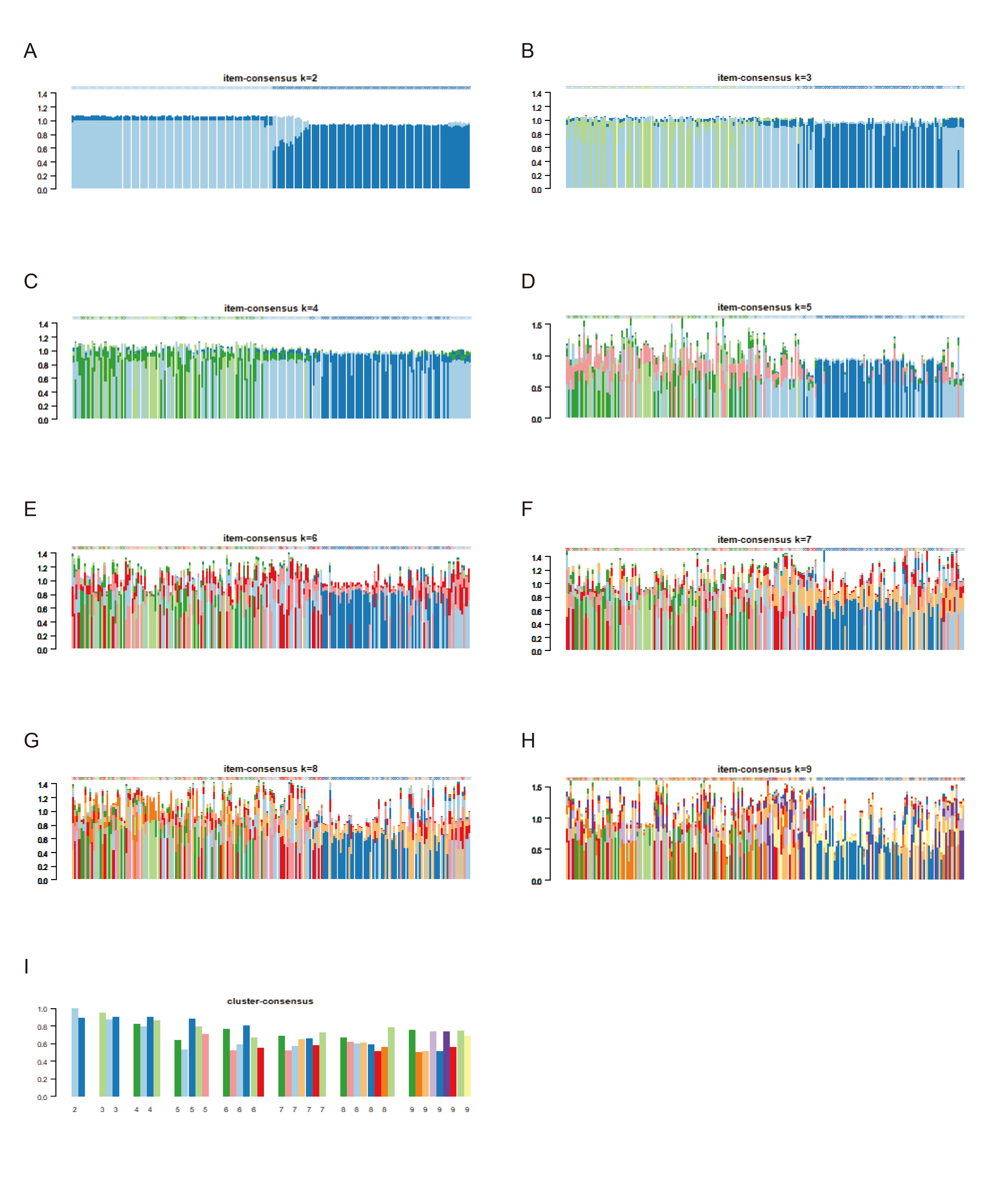


**Supplementary Fig.S3.** The item and score of consensus clustering when k = 2-9. (A) The item of consensus clustering when k = 2-4. (B) The item of consensus clustering when k = 5-7. (C) The item of consensus clustering when k = 8-9. (D) The score of consensus clustering when k = 2-9.


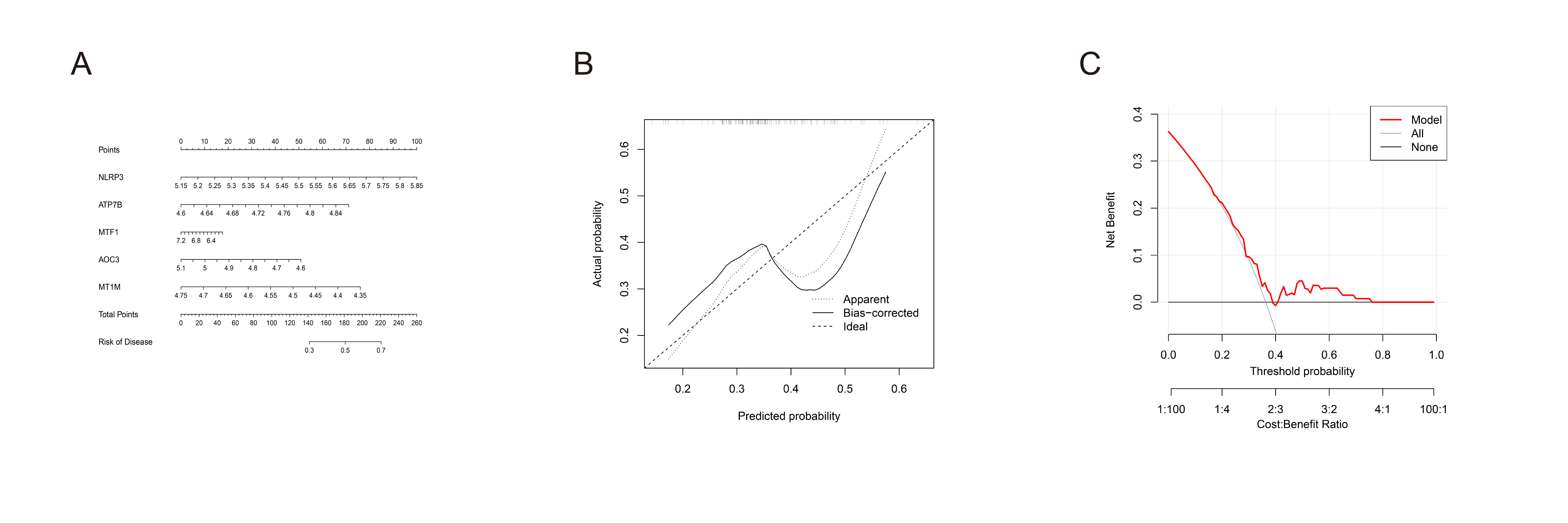


**Supplementary Fig.S4.** Validation of the value of nomogram in atherosclerosis diagnosis. (A) Nomogram of 5 A-CRGs. (B-C) Calibration curve and DCA of the nomogram.


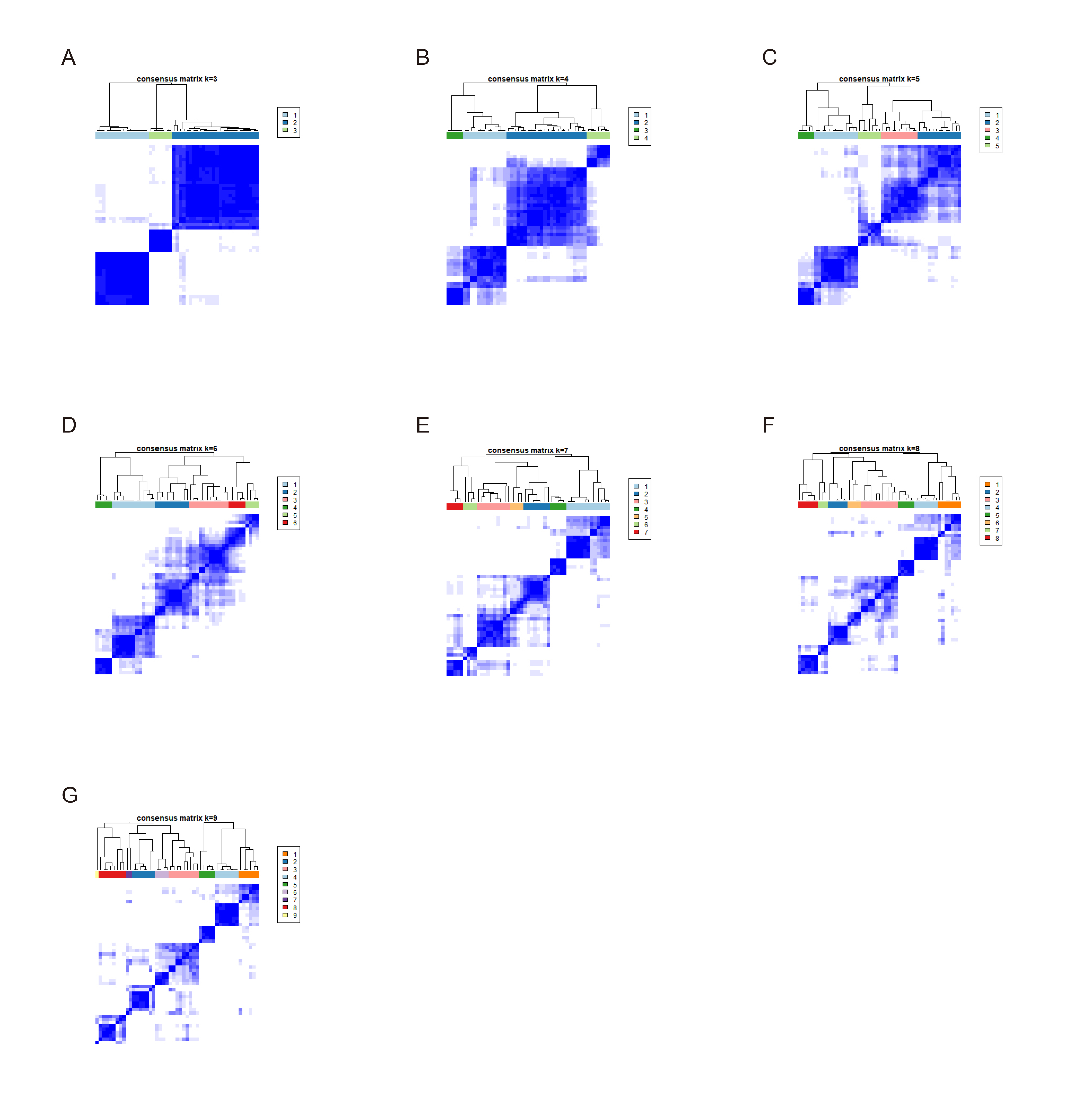


**Supplementary Fig.S5.** The consensus clustering matrix when k = 3-9 in the validation set. (A) k = 3. (B) k = 4. (C) k = 5. (D) k = 6. (E) k = 7. (F) k = 8. (G) k = 9.


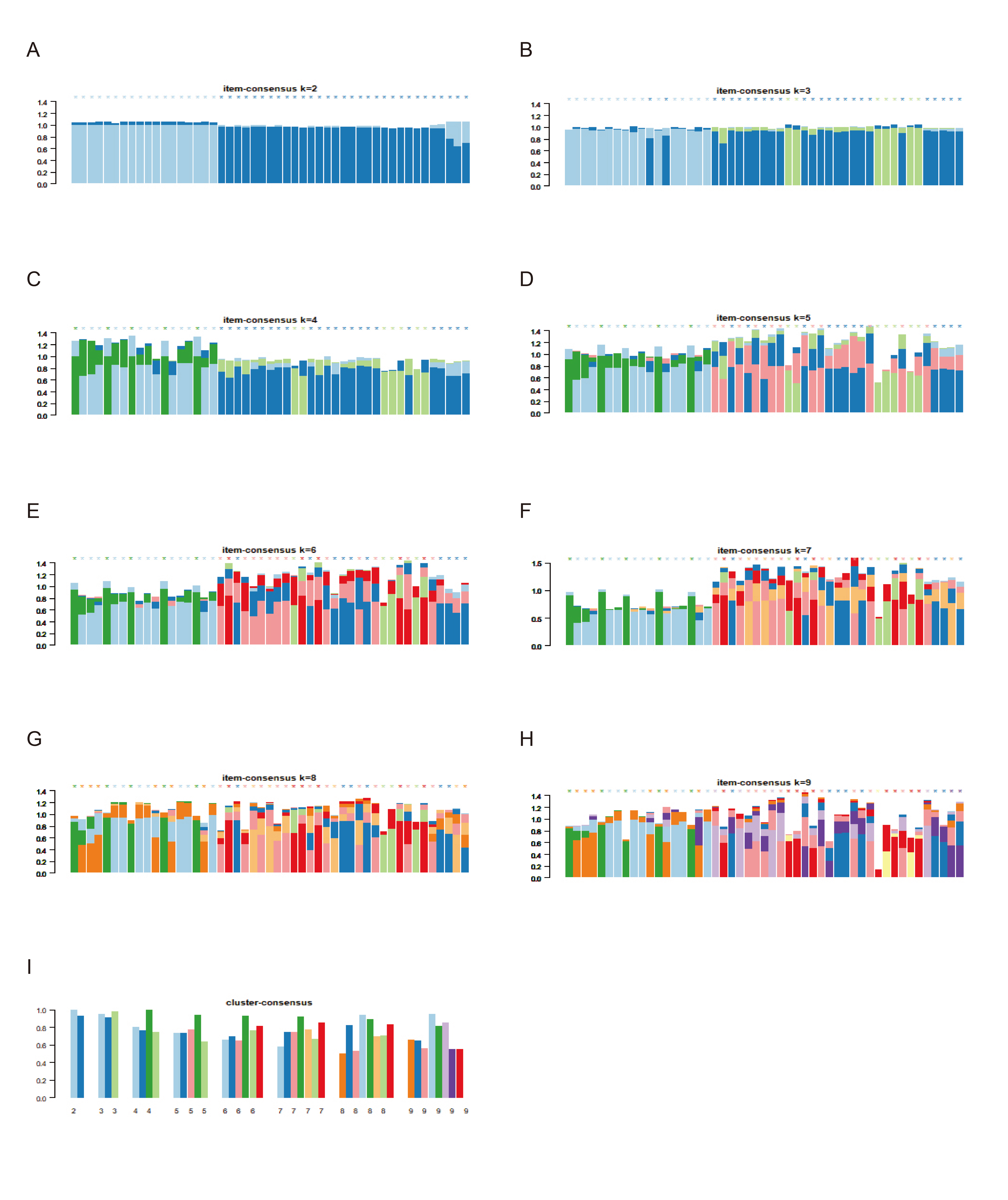


**Supplementary Fig.S6.** The item and score of consensus clustering when k = 2-9 in the validation set. (A) The item of consensus clustering when k = 2-4. (B) The item of consensus clustering when k = 5-7. (C) The item of consensus clustering when k = 8-9. (D) The score of consensus clustering when k = 2-9.


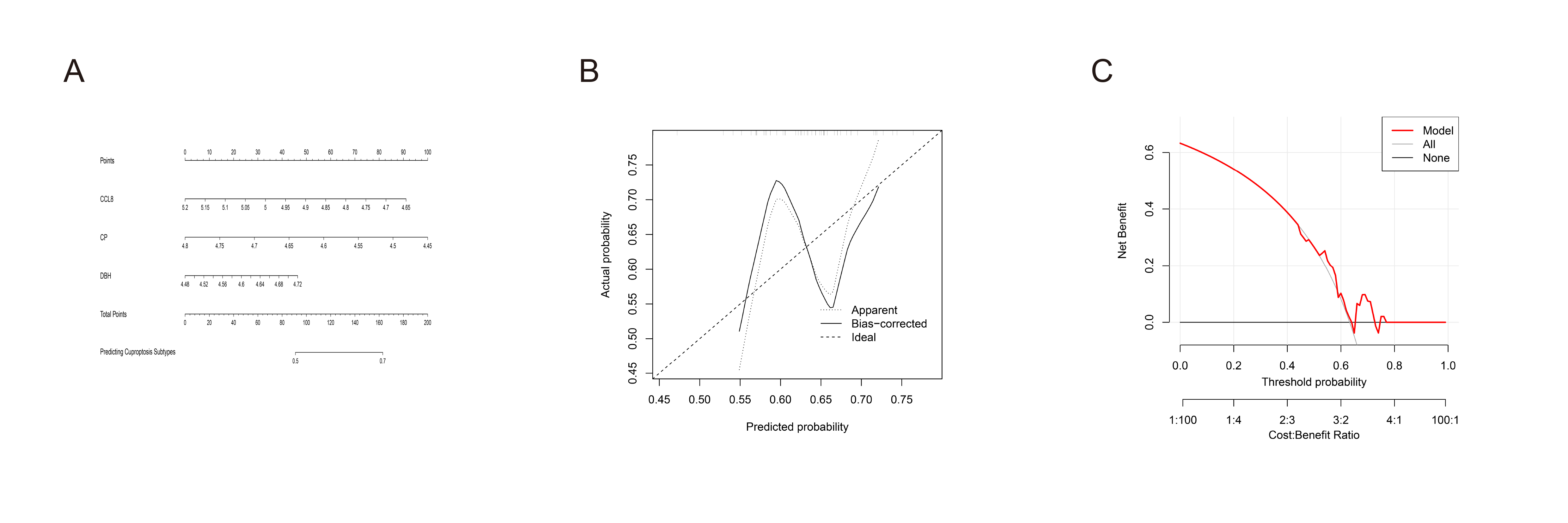


**Supplementary Fig.S7.** Validation of the value of nomogram in molecular subtypes identification. (A) Nomogram of 3 M-CRGs. (B-C) Calibration curve and DCA of the nomogram.


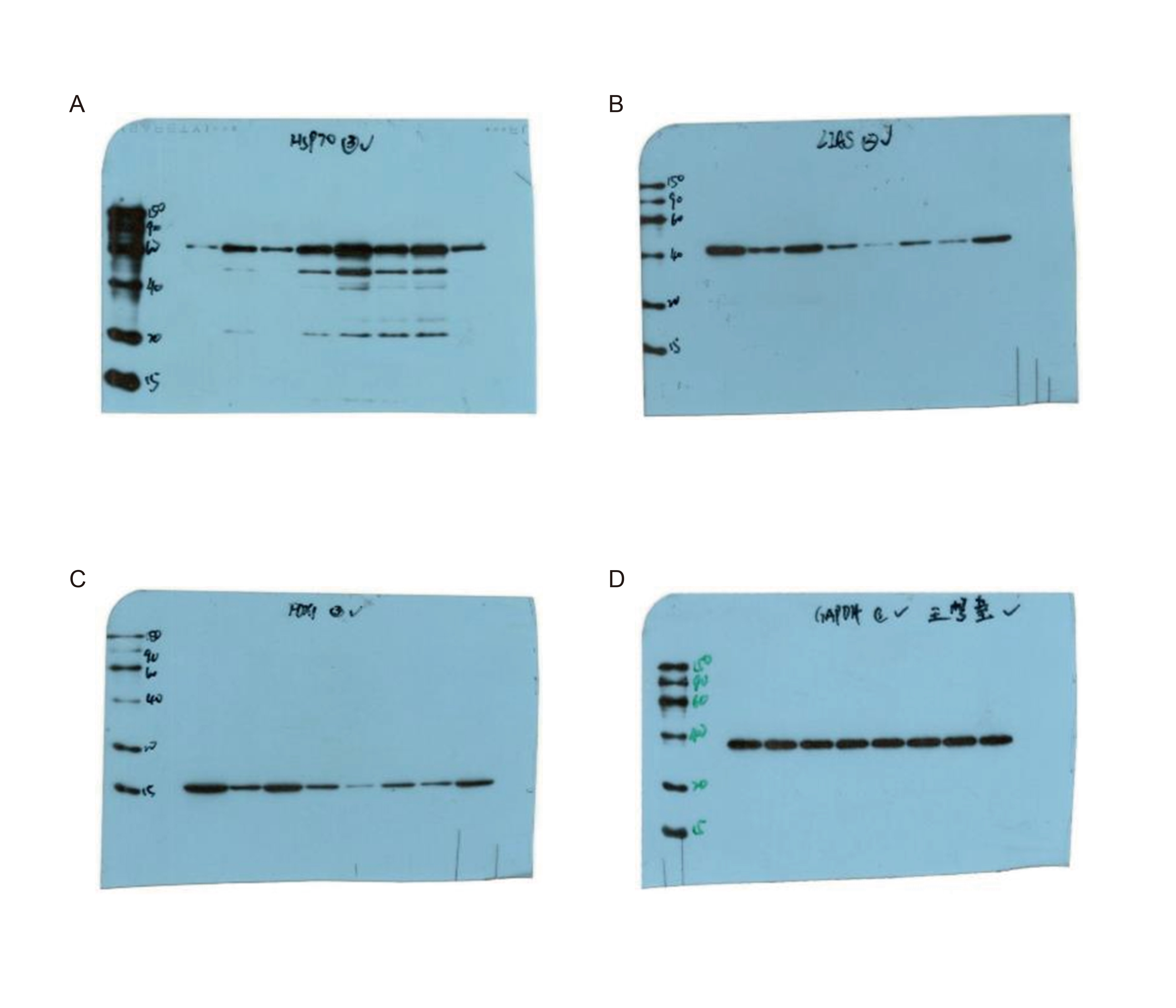


**Supplementary Fig.S8.** Uncropped images of western blot. (A) The uncropped image of HSP70. (B) The uncropped image of LIAS. (C) The uncropped image of FDX1. (D) The uncropped image of GAPDH.
